# A taxonomy of reliability indices for cluster‐level constructs measured by multiple individual‐level indicators

**DOI:** 10.1111/bmsp.70074

**Published:** 2026-10-14

**Authors:** Terrence D. Jorgensen, Suzanne Jak

**Affiliations:** ^1^ University of Amsterdam Amsterdam The Netherlands

**Keywords:** generalizability theory, inter‐rater reliability, intraclass correlation coefficient, multilevel structural equation model, scale reliability

## Abstract

Generalizability theory (GT) provides a variety of intraclass correlation coefficients (ICCs) to quantify reliability across different conditions of measurement—for example, scale and interrater reliability quantify generalization across items and raters, respectively. In a multilevel modelling (MLM) context, an ICC is often used to quantify the proportion of a subject‐level variable's variance that is attributable to cluster‐level differences (e.g., between schools or neighbourhoods). When data from cluster members are their ratings of a group‐level characteristic, the ICC is an inter‐rater reliability coefficient, interpreted as the degree to which observations of a cluster generalize across members of that cluster. Structural equation modelling (SEM) is becoming a popular framework for modelling GT, and the ‘omega’ indices of composite reliability—which are functions of SEM parameters—are special cases of GT (i.e., how measurements of a construct generalize across indicators). The merger of MLM and SEM (i.e., ML‐SEM) provides a unique opportunity to explore how well measurements of cluster‐level constructs generalize across multiple measurement conditions (indicators, cluster members, or both). A taxonomy of ICCs is explicated to appropriately quantify reliability across aspects of a procedure for measuring cluster‐level constructs, adding value to the limited ICCs currently considered in ML‐SEM applications.

Theories of human behaviour typically involve psychological constructs that cannot be directly observed (i.e., latent constructs). Multiple‐item scales or tests are typically used to capture individual differences due to a latent construct, where a scale composite (e.g., sum or average of scale items, number or proportion of correct test items) is used as an observed proxy for a latent variable. Classical test theory (CTT) has a long history of preoccupation with the reliability of such scale composites (Lord & Novick, 1968). Reliability represents the degree to which observed scores will be similar across repeated measurements (e.g., at different occasions, or using different tests) and is more formally defined in a later section. More modern test theories incorporate latent variables into statistical models—for example, person parameters in an item response theory (IRT) model, common factors in a confirmatory factor analysis (CFA) or structural equation model (SEM), or random effects in a linear mixed model (LMM). Modern measurement models for hypothesized latent constructs have enabled definitions of reliability to become more nuanced and for reliability coefficients to be estimated as functions of measurement‐model parameters.

This article focusses on Generalizability Theory (GT), which is a measurement model that can be estimated using LMM (Jiang, 2007; Putka et al., 2008) or SEM (Jorgensen, 2021; Marcoulides, 1996; Raykov & Marcoulides, 2006; Vispoel et al., 2018). We apply the GT framework to a particular measurement scenario that has raised some controversy in the past few years: measuring a group‐level construct using individual‐level data. This measurement scenario is often encountered in scientific fields that study people who are ‘clustered’ into many natural groups—for example, employees nested within departments or companies; patients nested within doctors or clinics; and students nested within teachers, classes, or schools. When the construct of interest refers to a trait about the group (e.g., school climate, group norms), individual members constitute multiple indicators of the group, which compounds with multiple scale items to yield many latent variables in the same model. Controversy exists even in how constructs are conceptually defined or labelled in multilevel settings, but we refer to group‐level constructs measured with error as ‘shared’ constructs (Jak et al., 2023; Stapleton et al., 2016; Stapleton & Johnson, 2019).

Many definitions of reliability have been proposed for multilevel data, some of which are directly relevant to the context discussed in this paper (Geldhof et al., 2014; Lai, 2021), which is when multiple cluster members serve as raters of their cluster’s construct and provide measurements using multiple scale items. In GT notation, this is denoted as a (r:c)×i design, where (r:c) represents the raters (r) nested in clusters (c), which are crossed with items (i). Other reliability coefficients have been defined for a highly analogous situation in which multiple item‐responses are replaced by multiple raters of the subjects nested in clusters—a (s:c)×r design (as well as for more complex designs that fall beyond our scope; Lin et al., 2025; Ten Hove et al., 2022). All of the cited proposals defined level‐specific scale‐composite reliability *or* inter‐rater reliability (IRR) coefficients for within‐level constructs, where the corresponding between‐level construct represents a cluster‐level aggregation of the individual‐level construct (i.e., a “configural” construct; Stapleton et al., 2016; Jak et al., 2023). There is generally consensus about how to define and interpret reliability coefficients for variables that represent Level‐1 (or within‐level) information but controversy about higher‐level variables. Our paper focuses on measuring shared constructs, which are inherently group‐level traits, so within‐level variability represents a specific dimension of error rather than true scores.

When measuring group‐level constructs with responses of group members to multiple‐item scales, we argue that researchers should evaluate the degree to which both sources of error (raters and items)—both jointly and separately—can prevent accurately distinguishing between groups. Although Lai (2021) proposed reliability coefficients relevant for this situation that jointly consider both sources of error, he recommended it instead for configural constructs, proposing a very similar coefficient (but using a different model) for shared constructs. To our knowledge, the only source that brings together all relevant reliability indices for this scenario under a single umbrella is a somewhat obscure section in an authoritative GT textbook by Brennan (2001, section 4.4.4, equations 4.31–4.33). His motivating example indeed involved class‐level constructs (teacher quality, provided by multiple students' responses to multiple questions), and he defined coefficients to quantify the degree to which measurements generalize over students, items, and both. Apparently overlooked even among the recent articles proposing higher‐level coefficients using GT (Lin et al., 2025; Ten Hove et al., 2022), Brennan's (2001, pp. 130–132) brief example provides insufficient guidance on its own for this relatively common context.

The primary goal of this article was to provide a more thorough introduction to useful reliability indices when measuring cluster‐level constructs with multiple scale items, so readers can understand how to address important research goals—namely, to critically evaluate both dimensions of their measurement procedure in order to reveal the most efficient way(s) to minimize measurement error. To fulfil this goal, we will (a) show how the GT framework can elucidate relationships among existing reliability coefficients defined for this or similar contexts (though often given different labels), (b) develop a comprehensive notation to report such reliability coefficients, and (c) provide guidance about how to interpret and utilize such coefficients. The article begins with a motivating example^1^ from education science. The principles of GT are introduced, focusing on decomposition of variance components that are then used to define various reliability coefficients. To facilitate interpretation of the coefficients defined using GT, we provide complementary definitions using the ‘correlation approach’ (Lin et al., 2025; Vangeneugden et al., 2005), which relies on covariance algebra to derive the same coefficients as (un)conditional correlations between repeated measurements of the same target. Using the motivating example data, we demonstrate how LMM and SEM can be used to estimate the variance components and reliability coefficients. We also illustrate how the coefficients can be used to inform how measurements can be made more reliable. We discuss how various previously proposed and commonly reported coefficients fit into the comprehensive GT framework, and we provide advice for researchers interested in validating scales used to measure cluster‐level constructs.

## 1 MOTIVATING EXAMPLE

Ye et al. (2024) evaluated the measurement of a dimension of school climate: school emphasis on academic success (SEAS). They analysed data available from the Trends in International Mathematics and Science Study (TIMSS; Mullis et al., 2020), provided with their supplementary materials on the associated Open Science Framework (OSF) project (Ye et al., 2022). Their goal was to compare measurements of the same construct from different sources (teachers or school principles) using 11 items that reference different members of the school (students, parents, or teachers). To avoid complications unrelated to this article’s goals, we focus only on parental SEAS, measured by teacher responses to four of the 11 items on a 1 (*very high*) to 5 (*very low*) scale. An example item is ‘How would you characterize parental expectations for student achievement within your school?’ Complete descriptions of all items can be found in Ye et al. (2024, table 1), but our examples focus only on Items 5–8. Ye et al. (2024) evaluated the measurement instrument using multilevel CFA. Figure 1 shows a path diagram depicting such a CFA for the 4‐indicator, 1‐factor model relevant to the subset of parental SEAS items (as measured by teachers). The parameters depicted in Figure 1 will be introduced with the corresponding structural equations in a later section.

**FIGURE 1:**
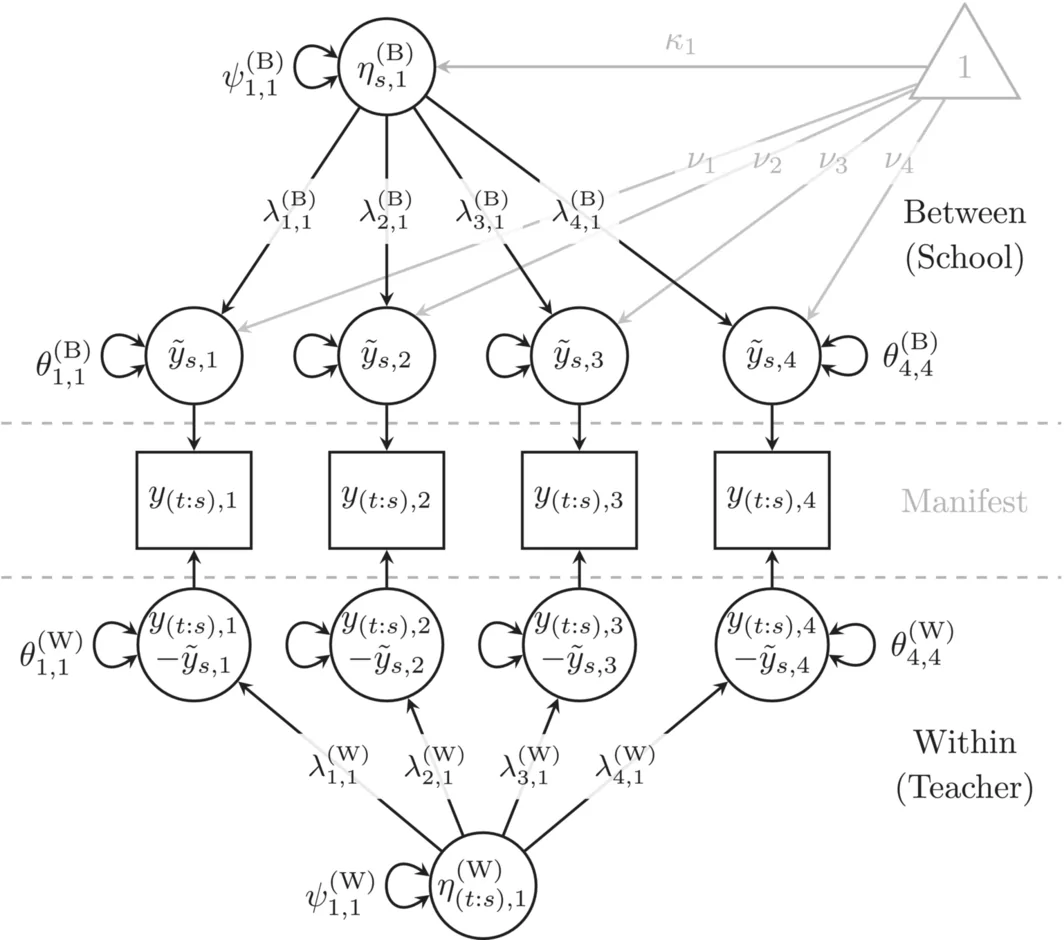
Path diagram depicting a multilevel CFA with 4 indicators (items) decomposed into latent school‐ (${\tilde{y}}_{s}$) and teacher‐level components ($y_{t:s}-{\tilde{y}}_{s}$), each measuring a single factor at that level.

SEAS is a school‐level construct, which is measured by perceptions of teachers within each school. Thus, SEAS is a shared construct (Jak et al., 2023), measured using both multiple items and multiple raters (teachers) per school. The construct of interest is therefore $\eta^{\left(\text{B}\right)}$ in Figure 1, a proxy of which might be calculated to include in subsequent analyses or utilize for decision‐making. For example, a school‐level composite (${\bar{y}}_{s}$) could be calculated to rank‐order schools in terms of their parental SEAS or to predict a school‐level outcome variable in a regression model:

(1) $$z_{s}=\beta_{0}+\beta_{z|y}{\bar{y}}_{s}+\varepsilon_{s}$$

such as school‐average student achievement, as analysed by Ye et al. 2024 using multilevel SEM (ML‐SEM). The school‐level composite for each School s could be calculated by taking the arithmetic mean ${\bar{y}}_{s}$ of responses $y_{(t:s)i}$ to Items i=1,…,4 by Teachers $t=1,\text{…},n_{t:s}$ within School s:

(2) $${\bar{y}}_{s}=\frac{1}{4\times n_{t:s}}\overset{4}{\underset{i=1}{\sum }}\overset{n_{t:s}}{\underset{t=1}{\sum }}y_{(t:s)i},$$

where $n_{t:s}$ is the number of teachers who rated parental SEAS in School s. The reliability $\rho_{y}$ of this composite has practical importance due to downstream consequences of how it is used to make decisions (e.g., draw inferences). For example, it quantifies the degree to which a simple‐regression coefficient (e.g., $\beta_{z|y}$ in Equation 1) would be attenuated due to measurement error, relative to the simple‐regression coefficient $\beta_{T}$ when regressing $z_{s}$ on an error‐free school‐level predictor $\mu_{s}$ (McDonald, 1999, p. 135, equation 7.33):

(3) $$\beta_{z|y}=\beta_{T}\sqrt{\rho_{y}}\;\Leftrightarrow \;\beta_{T}=\frac{\beta_{z|y}}{\sqrt{\rho_{y}}}.$$

The same form holds for correcting a correlation coefficient $\rho_{yz}$, which is equivalent to a standardized simple‐regression coefficient (McDonald, 1999, pp. 133–134, equations 7.23 and 7.25). The formula can be extended to correct the correlation for attenuation by measurement error also present in $z_{s}$ by dividing by (the square‐root of the product of) both variables’ reliabilities (McDonald, 1999, pp. 133–134, equations 7.24 and 7.26); however, correcting multiple‐regression coefficients for attenuation becomes more complicated. Note that these principles are also applicable to between‐level coefficients of multilevel models (e.g., cluster‐randomized trials; S.‐J. Cho & Preacher, 2016, pp. 775–778).

The school‐level composite in Equation (2) contains multiple sources of measurement error, complicating how to conceive of its reliability from a CTT perspective. Geldhof et al. (2014) proposed resolving this issue by simply applying CTT reliability coefficients (α, ω) to the latent components at each level of analysis in Figure 1. However, as Lai (2021) aptly criticized, Geldhof et al.'s (2014) level‐specific reliability coefficients do not reflect the reliability of the composite in Equation (2); instead, Geldhof et al.'s (2014) coefficients reflect hypothetical reliability of a composite that cannot be calculated from the unobserved school‐level components:

(4) $${\tilde{y}}_{s}=\overset{4}{\underset{i=1}{\sum }}{\tilde{y}}_{s,i},$$

where ${\tilde{y}}_{s,i}={𝔼}_{T}\left(y_{(t:s)i}\right)$ is the unobserved expected value (i.e., population mean) of teacher responses to Item i in School s, and the expectation ${𝔼}_{T}(.)$ is taken over the population of teachers (T, following notation specified in Lin et al., 2025, appendix A). Lai (2021) defined reliability coefficients that validly apply to observed‐data composites. Although he derived coefficients from a CTT perspective, we find that many of Lai's (2021) coefficients (which we will discuss in this paper) can be understood quite informatively from a GT perspective, and are analogous to those proposed by Brennan (2001, equations 4.31–4.33) but defined using CFA parameters and estimated under less restrictive assumptions.

## 2 GENERALIZABILITY THEORY

Whereas CTT only differentiates between true‐score variance and measurement‐error variance (Lord & Novick, 1968), GT extends CTT by allowing for variance to be decomposed between multiple sources of systematic and random error that arise from different design ‘facets’ (Brennan, 2001; Cronbach et al., 1963). The target of the measurement is the facet of *differentiation*, whose ‘universe scores’ are analogous to true scores in CTT. A given design for obtaining measurements of the target yields a universe of admissible observations that are contaminated with measurement error arising from the facets of *generalization* (e.g., multiple scale items, raters, or occasions). A generalizability coefficient quantifies the degree to which a single observed score—or a composite of $n_{\text{facet}}$ measurements of the same target—can be generalized across measurement scenarios, analogous to a reliability coefficient in CTT. Here, we focus only on the design described in our motivating example. For more thorough introductions to GT, we refer to Brennan (2010) and Vispoel et al. (2018), and for more exhaustive coverage of GT we refer to Brennan (2001), Cronbach et al. (1972), and Shavelson and Webb (1991).

For the SEAS construct, the target of measurement is the school, whose SEAS measurements differ due to perspectives of different teachers and how the different scale items are interpreted. Because each teacher only provides measurements of one school, these facets are nested (represented symbolically as t:s). Because every teacher (within every school) responds to the same four items, the item facet is crossed with the other facets, yielding a (t:s)×i design. A linear GT model for this design decomposes observed scores into variations around a grand mean μ:

(5) $$y_{(t:s)i}=\mu +\beta_{s}+\beta_{i}+\beta_{si}+\beta_{t:s}+\beta_{(t:s)i,\epsilon },$$

including the main effects of school ($\beta_{s}=\mu_{s}-\mu$) and items ($\beta_{i}=\mu_{i}-\mu$), their interaction ($\beta_{si}=\mu_{si}-\mu_{s}-\mu_{i}+\mu$), and the main effect of teachers ($\beta_{t:s}$, which is confounded with any teacher × school interaction due to nesting). The teacher (within school) × item interaction ($\beta_{(t:s)i,\epsilon }$) is likewise confounded with the three‐way interaction; furthermore, this is the highest‐order term in the model, so it is also confounded with random measurement error (ϵ), as indicated by the subscript. Every facet’s effect has an expected value of zero: 𝔼(β)=0.

### 2.1 Variance decomposition

Following from the assumption that Equation (5) is a linear combination of independent components with finite variance, the variance of observed scores can be decomposed into independent variance components associated with each component in Equation (5):

(6) $$\begin{array}{ll} \mathrm{Var}(y_{(t:s)i}) & =\mathrm{Var}(\beta_{s})\;+\;\mathrm{Var}(\beta_{i})\;+\;\mathrm{Var}(\beta_{si})\;+\;\mathrm{Var}(\beta_{t:s})\;+\;\mathrm{Var}(\beta_{(t:s)i,\epsilon }),\; \end{array}$$

(7) $$\begin{array}{ll} \sigma_{y}^{2} & =\sigma_{s}^{2}\;+\;\sigma_{i}^{2}\;+\;\sigma_{si}^{2}\;+\;\sigma_{t:s}^{2}\;+\;\sigma_{(t:s)i,\epsilon }^{2}.\; \end{array}$$

Whereas the subscripts in Equations 5 and 6 indicate the dimension(s) across which each effect varies, the subscripts in Equation (7) merely identify a variance component, which is typically^2^ assumed to be constant across units sampled from that dimension.

Although the linear model in Equation (5) does not require facet effects to follow a specific distribution, practical applications often involve assuming they are normally distributed. For ease of presentation, we therefore use the normal‐distribution's variance parameter $\sigma^{2}$ (Equation 7) in place of the generic Var(*) operator (Equation 6) when defining coefficients in the following subsections.

### 2.2 Relative and absolute error

Observations from a GT design can be used to compare scores between subjects/objects from the facet of differentiation, but such comparisons are complicated by measurement error. GT distinguishes between relative and absolute error. *Relative error* is relevant when making norm‐based decisions (e.g., comparing 2 schools' relative standing, regardless of how high or low their actual scores are), whereas *absolute error* is relevant when making criterion‐referenced decisions (e.g., comparing a school's score to a specified threshold; Vispoel et al., 2018). McGraw and Wong (1996) referred to indices of *consistency* and *agreement* as being defined using relative and absolute error, respectively, which are also called generalizability and dependability coefficients, respectively (Vispoel et al., 2018).

From the motivating example's (t:s)×i design, observed scores $y_{(t:s)i}$ and $y_{(t^{\prime }:s^{\prime })i^{\prime }}$ can differ due to any effects in Equation (5) that include the s subscript, not only to ‘true’ differences between schools. The shared SEAS construct’s *universe scores* (the GT analogue of true scores in CTT) are represented in Equation (5) as the sum of the grand mean μ with the school effect $\beta_{s}$, which captures school‐specific differences that cannot be attributed to differences between teachers' (i.e., raters') perceptions of the school ($\beta_{t:s}$), nor to how items are interpreted differently between individual teachers ($\beta_{(t:s)i,\epsilon }$) or on average between schools ($\beta_{si}$). All variance components that include the target's (i.e., school) subscript contribute to the relative error term: $\sigma_{si}^{2}+\sigma_{t:s}^{2}+\sigma_{(t:s)i,\epsilon }^{2}$.

Item‐specific factors ($\beta_{i}$) can make School's scores higher or lower, depending on the item(s) used, but they do not affect the rank‐ordering of schools. Thus, its variance component ($\sigma_{i}^{2}$) is omitted from indices of consistency but included in the absolute error term to define indices of agreement (McGraw & Wong, 1996; Vispoel et al., 2018). If teachers rated all schools rather than only their own (i.e., a crossed rather than nested facet), then teacher‐specific factors would not affect rank ordering of schools. However, due to the nesting of teachers within schools, the confounded variance component $\sigma_{t:s}^{2}=\sigma_{t}^{2}+\sigma_{ts}^{2}$ means that the absolute‐error component $\sigma_{t}^{2}$ cannot be disentangled from the relative‐error component $\sigma_{ts}^{2}$ (Brennan, 2001). This led McGraw and Wong (1996) to drop the consistency–agreement distinction from their notation for indices of nested (one‐way model) designs.

### 2.3 Random versus fixed facets

Although the number of teachers in a school is finite in practice, the teachers who are selected to respond to the SEAS scale constitute a random sample of teachers from that school, just as the schools constitute a random sample of schools sampled for the TIMSS project (Mullis et al., 2020). One can also argue that these four items are a random sample from a population of possible items to include in the measurement tool, in which case the measurement properties only differ due to sampling error, so reliability coefficients proposed in the next section reflect the consistency (or agreement) expected of scores measured using other teachers and items.

However, the TIMSS project (Mullis et al., 2020) does not randomly sample new items to measure SEAS when it samples new schools and teachers every 4 years. Thus, these $n_{i}=4$ items could instead be considered a *fixed* facet, so generalizations are only expected to apply to new samples that use the same items. We adopt the convention of Brennan (2001, p. 59) by using a capital $N_{i}\ll \infty$ to indicate when we consider our items to be the entire ‘population’ of items used for measurement. Treating these $N_{i}=4$ items as a fixed facet has consequences for how the reliability coefficients defined in the next section for random effects must be adapted (compare Brennan, 2001, figure 4.4 for a fixed facet on p. 123 to figure 4.1 on p. 106 when the same facet is random).

Although Shrout and Fleiss (1979) and McGraw and Wong (1996) discussed ICCs for fixed raters, Ten Hove et al. (2024, p. 973) argued it is difficult to defend in research settings where raters are unlikely to remain constant across studies. Likewise, we do not think it is reasonable to consider lower‐level units (here, teachers) as fixed when measuring cluster‐level constructs because generalizability is meant to apply to any new (random) sample of the target facet (here, schools). It follows that in such a multistage sampling design, sampling new clusters would imply that in practice, new cluster members must also be sampled within those clusters. Nonetheless, an index of generalizability *only* over scale items implicitly considers the nested facet fixed (Brennan, 2001, section 4.4.4), but we discuss its interpretation after defining that index. We do, however, explicitly consider the case of fixed items in the following section, which is more realistic in the design of our motivating example.

## 3 INTRACLASS CORRELATION COEFFICIENTS FOR A SHARED CONSTRUCT

In CTT, the classic formula for reliability expresses the ratio of true‐score variance ($\sigma_{T}^{2}$) to total variance ($\sigma_{y}^{2}$) of an observable variable y:

(8) $$\rho_{y,y^{\prime }}=\rho_{y,T}^{2}=\frac{\sigma_{T}^{2}}{\sigma_{T}^{2}+\sigma_{E}^{2}}=\frac{\sigma_{T}^{2}}{\sigma_{y}^{2}},$$

where $\sigma_{E}^{2}$ is composed of all measurement‐error variance. This can be described as an intraclass correlation coefficient (ICC), which is equivalently interpreted as the squared correlation between true and observed scores. The commonly used symbol $\rho_{y,y^{\prime }}$ follows from its interpretation as the correlation between parallel observed scores y and $y^{\prime }$. The ICC has long been used as a reliability coefficient in CTT (Bartko, 1966), particularly to quantify IRR (Gwet, 2021; McGraw & Wong, 1996; Shrout & Fleiss, 1979; Ten Hove et al., 2022).

Reliability coefficients in GT are also ICCs, but a greater variety of definitions follow from the distinction between relative and absolute error, decomposition of error into multiple components, and whether to quantify reliability of individual or composite (e.g., averaged) measurements. A nomenclature introduced by Shrout and Fleiss (1979) was improved by McGraw and Wong (1996) to report ICCs in a format that indicates these varieties. However, the conventions were only developed for single‐facet designs that capture a single source of measurement error. For example, ICC(1) reflects the reliability of single scores in a nested design, whereas ICC(k) reflects the reliability of composite scores calculated by averaging over k items, raters, etc.—for example, ICC(4) would indicate a composite that averaged over k=4 measurements. For crossed‐facet designs, ICC(C,k) reflects consistency of composite scores (or single scores when k=1), whereas ICC(A,k) reflects absolute agreement among composite scores.

The more complex (t:s)×i design of our motivating example necessitates extending the current convention to accommodate more facets. When defining reliability coefficients in the following sections, we will replace k with more immediately identifiable symbols corresponding to the number of measurements *per facet of generalization*. For example, ${\text{ICC}}_{s}(\text{C},n_{t:s}=20,n_{i}=1)$ would represent the consistency (indicated by ‘C’) of school scores (facet of differentiation indicated by subscript s) calculated by averaging across $n_{t:s}=20$ teachers per school, each of whom would respond to a single item ($n_{i}=1$). Some of the ICCs we define in upcoming sections have more complex subscripts because they apply to joint facets of differentiation, such as teachers within schools (t:s) or item responses about schools (si).

### 3.1 Generalizability and dependability with random facets

We first define ICCs representing generalizability and dependability, considering both facets as random. We then discuss implications of fixed facets to define a coefficient of IRR^3^ conditional on fixed items, followed by an inter‐item^4^ reliability coefficient conditional on fixed raters, as originally proposed by Brennan (2001, section 4.4.4). To illustrate the connections and distinctions among these coefficients (and others discussed in a later section on Connections with Past Research), Table 1 lists each index (per row) and indicates which variance components from Equation (7) appear in its denominator.

**TABLE 1:** Role of each variance component in ICC definitions for shared constructs.

		Generalizes over …		Denominator includes				
ICC	Equation(s) in paper	Items	Raters	$\sigma_{i}^{2}$	$\sigma_{si}^{2}$	$\sigma_{t:s}^{2}$	$\sigma_{(t:s)i,\epsilon }^{2}$	Other notations
${\text{ICC}}_{s}(\text{C},n_{t:s},n_{i})$	9	✓	✓		✓	✓	✓	G‐coef, $𝔼(\rho^{2})$, $\alpha^{\text{b}}$, $\omega^{\text{b}}$
${\text{ICC}}_{s}(\text{A},n_{t:s},n_{i})$	11	✓	✓	✓	✓	✓	✓	D‐coef, Φ
${\text{ICC}}_{si}(\text{A},n_{t:s},N_{i})$	14		✓		✓ ^a^	✓	✓	
${\text{ICC}}_{t:s}(\text{C},n_{t:s},n_{i})$	16	✓			✓	✓ ^a^	✓	$\alpha^{\text{2l}}$, $\omega^{\text{2l}}$ (when $n_{t:s}=1$)
${\text{ICC}}_{t:s}(\text{A},n_{t:s},n_{i})$	18	✓		✓	✓	✓ ^a^	✓	
${\text{ICC}}_{\text{O}}$	(40), (41), (42)		✓			^b^		ICC(1,1) ^c^, ICC(1)^d^
$\text{ICC}(n_{t:s})$	46		✓			^b^		ICC(1,k) ^c^, ICC(k)^d^
${\text{ICC}}_{\text{L}}$	(41), (44)		✓			✓		
^e^	45	✓			✓			${\tilde{\alpha }}^{\text{b}}$, ${\tilde{\omega }}^{\text{b}}$

*Note*: ICC = intraclass correlation coefficient, α implies the fitted model assumes parallel (or at least tau‐equivalent) items, ω implies the model allows items to be congeneric. The top section (2 rows) contains coefficients that generalize over both dimensions of error; the middle section (5 rows) contains reliability of an observed (composite of) variable(s) over only one dimension of error; the bottom section (2 rows) contains hypothetical reliability of a latent variable or unobservable composite.

^a^ Component also included in numerator.

^b^ These are single‐item IRR coefficients, so an analogous variance component would be included from a univariate model.

^c^ Shrout and Fleiss (1979).

^d^ McGraw and Wong (1996), who both used k instead of $n_{t:s}$.

^e^ Our paper does not explicitly define indices proposed by Geldhof et al. (2014), which Lai (2021) denoted with ${\tilde{\alpha }}^{\text{b}}$ and ${\tilde{\omega }}^{\text{b}}$ to contrast with his $\alpha^{\text{b}}$ and $\omega^{\text{b}}$.

#### 3.1.1 Generalizability

A generalizability coefficient (G‐coef) expresses the target variance proportional to relative error (the parenthetical term in the denominator):

(9) $${\text{ICC}}_{s}(\text{C},n_{t:s},n_{i})=\frac{\sigma_{s}^{2}}{\sigma_{s}^{2}+\left(\frac{\sigma_{t:s}^{2}}{n_{t:s}}+\frac{\sigma_{si}^{2}}{n_{i}}+\frac{\sigma_{(t:s)i,\epsilon }^{2}}{n_{t:s}\times n_{i}}\right)},$$

where the highest‐order variance term ($\sigma_{(t:s)i,\epsilon }^{2}$) is reduced by averaging over $n_{t:s}\times n_{i}$ measurements. This is equivalent to Brennan (2001, equation 4.31), and its interpretation as a ‘generalizability’ coefficient follows from it quantifying the degree to which a composite of $n_{t:s}$ teachers’ responses to $n_{i}$ items can be generalized to a composite of $n_{t:s}$ different teachers' responses to $n_{i}$ different items, each sampled from the same populations of teachers (from the same schools) and of items, respectively. In other words, how correlated could one expect the rank‐ordering of schools to be with a composite using $n_{t:s}$ different teachers and $n_{i}$ different items?

Consistency across averaged scores corresponds to the CTT internal‐consistency reliability coefficient α (Cronbach, 1951; Sijtsma, 2009), indicating the expected squared correlation $𝔼(\rho^{2})$ between a school's composite and its true (universe) score. The G‐coef is a conditional correlation because average differences between items are partialed out by omitting $\sigma_{i}^{2}$ from Equation (9). By setting $n_{t:s}=n_{i}=1$ in Equation (9), we obtain the degree to which a single Teacher t's response to Item i about School s can be generalized to the response of a different teacher (from the same school) to a different item. Thus, ${\text{ICC}}_{s}(\text{C},n_{t:s}=1,n_{i}=1)$ is the squared (conditional) correlation between a school's single score ($y_{(t:s)i}$) and its universe score, or the correlation between parallel single measurements ($y_{(t:s)i}$ and $y_{(t^{\prime }:s)i^{\prime }}$). In the notation used by Vangeneugden et al. (2005) and Lin et al. (2025) for the correlation approach:

(10) $$\begin{array}{cc} {\text{ICC}}_{s}(\text{C},n_{t:s}=1,n_{i}=1) & =\text{Cor}(y_{(t:s)i},y_{(t^{\prime }:s)i^{\prime }}\;|\;i,i^{\prime })\\ \left(\text{only when}\;\sigma_{t}^{2}=0\right) & =\text{Cor}(y_{(t:s)i},y_{(t^{\prime }:s)i^{\prime }}\;|\;i,i^{\prime },t,t^{\prime }). \end{array}$$

It bears repeating that this design does not permit separating relative from absolute teacher error because $\sigma_{t}^{2}$ and $\sigma_{ts}^{2}$ are confounded in the nested component's $\sigma_{t:s}^{2}$. Thus, except when $\sigma_{t}^{2}=0$ (which cannot be verified when this facet is nested), Equation (9) simultaneously quantifies consistency across items and agreement across teachers. Hence, the correlation in Equation (10) can only condition on items in practice, not on teachers (see Lin et al., 2025, equation 19).

#### 3.1.2 Dependability

Consistency indices are appropriate to quantify the degree to which a correlation or unreliable predictor's simple‐regression coefficient is attenuated due to measurement error (Cho & Preacher, 2016). Such coefficients only concern how individuals are rank‐ordered and are not affected by centring the variables higher or lower on the number line (as might occur when using more ‘difficult’ items or more ‘strict’ raters). When scores are instead compared to an absolute numeric criterion (e.g., when used to classify the targets of measurement), then absolute error enters the ICC definition (compare the first 2 rows of Table 1). A dependability coefficient (D‐coef) includes the variability of items on average ($\sigma_{i}^{2}$), as well as the degree to which a numeric classification criterion^5^ ξ differs from the mean μ:

(11) $${\text{ICC}}_{s}(\text{A},n_{t:s},n_{i})=\frac{\sigma_{s}^{2}+{\left(\xi -\mu \right)}^{2}}{\sigma_{s}^{2}+{\left(\xi -\mu \right)}^{2}+\left(\frac{\sigma_{t:s}^{2}}{n_{t:s}}+\frac{\sigma_{i}^{2}+\sigma_{si}^{2}}{n_{i}}+\frac{\sigma_{(t:s)i,\epsilon }^{2}}{n_{t:s}\times n_{i}}\right)}.$$

A global^6^ D‐coef (often denoted Φ in GT literature, e.g., Brennan, 2010, equation 13) is obtained by setting ξ=μ in Equation (11), which corresponds to a (no longer conditional) correlation between parallel composites, or between parallel measurements when setting $n_{t:s}=n_{i}=1$:

(12) $${\text{ICC}}_{s}(\text{A},n_{t:s}=1,n_{i}=1)=\text{Cor}(y_{(t:s)i},y_{(t^{\prime }:s)i^{\prime }}),$$

using notation from the correlation approach (Lin et al., 2025; Vangeneugden et al., 2005).

#### 3.1.3 Estimating ICCs for future data

Point estimates of Equations (9) and (11) can be obtained by plugging in estimates of the relevant variance components. The sample sizes of each design facet need not correspond to the same design employed to estimate the variance components. Given unbiased estimates of the variance components in Equation (7), the reliability of hypothetical composites can be calculated for other designs^7^, to inform an optimal design for obtaining future measurements. In fact, a single sample size might not even apply to the observed data when unbalanced designs are used. In our SEAS example data, the number of teachers ($N_{t}=418$) per school ($N_{s}=156$) ranged from $n_{t:s}=1$ (44 schools) to 6 teachers (6 schools). In this case, a composite of the observed data involves averaging over a different number of teachers per school, so the reliability of that composite is calculated using the harmonic‐mean number of teachers per school $n_{t:s}$ (see also Putka et al., 2008, about ICCs for unbalanced crossed designs):

(13) $${ñ}_{t:s}={\left(\frac{1}{N_{s}}\overset{N_{s}}{\underset{s=1}{\sum }}n_{t:s}^{-1}\right)}^{-1},$$

which is ${ñ}_{t:s}=1.908$ in our sample. Substituting n=1 for either sample size in Equation (9) (or 11) quantifies how generalizable (or dependable) scores are expected to be across individual teachers or in response to a single item.

The advantage of the G‐coef is that it quantifies reliability—and its implications for attenuation—simultaneously across all sources of measurement error in the GT design. This paper focusses on a specific scenario in which a cluster‐level shared construct is measured using multiple‐item scales responded to by members of each cluster. It can also be informative to quantify both inter‐item reliability and IRR indices, each of which indicate the degree to which a measurement tool or protocol could be improved to yield better measurements. If improving the reliability of individual measurements is infeasible, reliability of a composite could also be improved by designing a future study that uses more raters or items. Given the relative costs of each possibility, an optimal design might be sought to yield a minimum acceptable reliability at the lowest possible cost.

### 3.2 Generalizability and dependability with fixed facets

When only a subset of multiple dimensions of error (i.e., facets of generalization) are to be generalized over, this can be seen as synonymous with considering the complementary subset of facets of generalization to be fixed (Brennan, 2001, section 4.4.4). We begin with IRR given fixed items, which allows us to discuss the implications of treating a facet as fixed. We then discuss the difficulties of considering nested raters as fixed, but nonetheless useful to define an index of inter‐item reliability to evaluate that dimension of the measurement design.

#### 3.2.1 Interrater reliability

The utility of quantifying IRR even in the presence of item‐specific error is that one can diagnose the degree to which unique rater perspectives contaminate item or composite scores. This allows researchers to consider the potential impact of reducing heterogeneity in rater perceptions, independent of considerations about inter‐item reliability. In our SEAS example, an IRR coefficient would be intended to quantify how measurements generalize across different teachers who respond to the scale, but not how scale responses would generalize to different items. Thus, item‐related error variance must be included in the numerator of the ICC, reflecting it as part of the expected universe‐score variance. Our ICC's subscript is therefore si to denote it as generalizability of school scores using these particular items. Given that teacher effects cannot be disentangled from the teacher × school interaction to distinguish relative from absolute error, we define this IRR index as an agreement index, since it certainly always reflects absolute agreement among raters (Brennan, 2001, section 4.4.4 also noted the equivalence of G‐ and D‐coefs for this index). Only under the restrictive assumption that $\sigma_{t}^{2}=0$ (which is empirically unverifiable and theoretically different to defend) would this IRR coefficient reflect consistency, but in that case, it could not be distinguished from absolute agreement anyway.

According to Brennan (2001, compare figure 4.4 for a fixed facet on p. 123 to figure 4.1 on p. 106 when the same facet is random), a fixed‐facet's main effect is removed from the total variance, whereas its interaction with the target effect becomes part of the universe‐score variance used in the numerator. Thus, treating our $N_{i}=4$ items as fixed has consequences for how the D‐coef in Equation (11) must be adapted. First, the denominator must *exclude* the variance component for fixed items ($\sigma_{i}^{2}$) because the same item effects will be present when different schools (and teachers within them) are sampled. Second, the numerator must now *include* the school × item interaction component ($\sigma_{si}^{2}$) as part of true‐score variance:

(14) $${\text{ICC}}_{si}(\text{A},n_{t:s},N_{i})=\frac{\sigma_{s}^{2}+\frac{\sigma_{si}^{2}}{N_{i}}}{\sigma_{s}^{2}+\frac{\sigma_{si}^{2}}{N_{i}}+\left(\frac{\sigma_{t:s}^{2}}{n_{t:s}}+\frac{\sigma_{(t:s)i,\epsilon }^{2}}{n_{t:s}\times N_{i}}\right)}.$$

This is a G‐coef that is equivalent to a D‐coef (Brennan, 2001, p. 130, equation 4.33) due to the nesting of raters (teachers) under the facet of differentiation (schools).

One may quantify the degree to which a single teacher's composite of item responses generalizes to another teacher's by setting $n_{t:s}=1$. Following the notation of Lin et al. (2025, appendix E), the correlation among composites of all fixed items i can be defined as:

(15) $${\text{ICC}}_{si}(\text{A},n_{t:s}=1,N_{i})=\text{Cor}(y_{(t:s)+},y_{(t^{\prime }:s)+}\;|\;\forall_{i}),$$

where the subscript ‘+’ indicates taking a sum or average across all items, and conditioning on all of them (represented by the ‘for all’ symbol: ∀). The interpretations differ between the interrater agreement coefficient in Equation 14 and the D‐coef in Equation (11), as reflected by comparing the rows of Table 1 corresponding to those equations. Equation (11) does not condition on items—thus treating items as random—so they reflect the degree to which a composite of $n_{t:s}$ teachers’ (sampled from the same school s) responses to *any randomly sampled set* of $n_{i}$ items can be generalized to a composite of $n_{t:s}$ *different* teachers' responses to *another set* of $n_{i}$ randomly sampled items. In other words, how correlated could one expect schools' scores to be, when scores are a composite of $n_{t:s}$ different teacher responses to different items?

In contrast, the IRR coefficient in Equation (14) conditions on items, thus treating them as fixed. So it reflects the degree to which a composite of $n_{t:s}$ teachers' responses to *these* $N_{i}=4$ items can be generalized to a composite of $n_{t:s}$ different teachers' responses to *the same* $N_{i}=4$ items, when sampled from the same school s.

If the teacher main effect were not confounded due to nesting, then its interpretation could be stated as how correlated one could expect the rank‐ordering of schools to be (i.e., consistency) with a composite using $n_{t:s}$ different teachers but the same $N_{i}=4$ items. However, although fixed items do not contribute to absolute error, average differences between teachers would affect rank‐ordering of schools, due to teachers being nested within schools.

#### 3.2.2 Inter‐item reliability

An index of inter‐item reliability can be defined similar to the G‐coef in Equation (9). However, a inter‐item reliability coefficient would not be intended to quantify how measurements generalize across different teachers who respond to the scale. Thus, teacher‐related relative^8^ error variance must be included also in the numerator of the ICC:

(16) $${\text{ICC}}_{t:s}(\text{C},n_{t:s},n_{i})=\frac{\sigma_{s}^{2}+\frac{\sigma_{t:s}^{2}}{n_{t:s}}}{\sigma_{s}^{2}+\frac{\sigma_{t:s}^{2}}{n_{t:s}}+\left(\frac{\sigma_{si}^{2}}{n_{i}}+\frac{\sigma_{(t:s)i,\epsilon }^{2}}{n_{t:s}\times n_{i}}\right)},$$

where the ICC subscript t:s indicates this coefficient's target of measurement is teachers within schools. Despite the target of measurement actually being clusters for a shared construct, the index in Equation (16) is only meant to quantify generalizability over items (i.e., inter‐item reliability, or internal consistency), not cluster members, as reflected in those rows of Table 1 (see also an equivalent definition in Brennan, 2001, equation 4.32). One can interpret this as the expected correlation between two measurements of the same randomly sampled school given by the same randomly sampled teacher within that school, but using two different items (Lin et al., 2025; Vangeneugden et al., 2005):

(17) $$\begin{array}{cc} {\text{ICC}}_{t:s}(\text{C},n_{t:s}=1,n_{i}=1) & =\text{Cor}(y_{(t:s)i},y_{(t:s)i^{\prime }}\;|\;t,i,i^{\prime })\\ \left(\text{due to nesting}\;\right) & =\text{Cor}(y_{(t:s)i},y_{(t:s)i^{\prime }}\;|\;i,i^{\prime }). \end{array}$$

Note that despite this coefficient implicitly treating teachers as fixed, we refrain from using a capital $N_{t:s}$ because we do not take seriously the assumption that teachers are actually fixed when gathering future samples. The practical purpose of this coefficient is merely to evaluate the degree to which measurements are contaminated specifically by item‐specific error.

Whereas the G‐coef reflects how reliably measurements can distinguish between clusters (schools)—jointly across cluster members (teachers) and items—the ICC in Equation (16) narrows this evaluation of reliability on a particular dimension of measurement error (items) so that researchers can consider how to adjust their measurement procedure or instrument in order to minimize that specific source of error.

One may quantify the degree to which individual responses (or a composite of $n_{t:s}\geq 2$ teachers' responses) to a single scale item generalize to another item by setting $n_{i}=1$ in Equation (16). Likewise, one may quantify the degree to which a single teacher's item response (or scale composite) generalizes to another (composite of) item(s) by setting $n_{t:s}=1$. One may also quantify absolute agreement between (composites of) items (i.e., not conditioning the correlation in Equation (17) on particular items i and $i^{\prime }$) by including the item‐effects variance component ($\sigma_{i}^{2}$) in the denominator, as it appears in Equation (11):

(18) $$\begin{array}{cc} {\text{ICC}}_{t:s}(\text{A},n_{t:s},n_{i}) & =\frac{\sigma_{s}^{2}+\frac{\sigma_{t:s}^{2}}{n_{t:s}}}{\sigma_{s}^{2}+\frac{\sigma_{t:s}^{2}}{n_{t:s}}+\left(\frac{\sigma_{i}^{2}+\sigma_{si}^{2}}{n_{i}}+\frac{\sigma_{(t:s)i,\epsilon }^{2}}{n_{t:s}\times n_{i}}\right)}\\ & =\text{Cor}(y_{(t:s)i},y_{(t:s)i^{\prime }}). \end{array}$$

The utility of quantifying inter‐item reliability even in the presence of rater error is that one can diagnose the degree to which item‐specific properties contaminate individual or composite scores. This allows researchers to consider the potential impact of reducing measurement error in their scales, independent of considerations about rater error.

## 4 ILLUSTRATION USING A LMM

Having defined reliability indices in the previous section, we use this section to report the results of an illustrative analysis in R (version 4.6.1; R Core Team, 2026), which readers can reproduce using the data and R‐syntax files available^9^ from our OSF project. We analysed the parental SEAS data (responses to $N_{i}=4$ items by $N_{t}=418$ teachers nested in $N_{s}=156$ schools) using the mixed‐modelling package lme4 (version 1.1‐38; Bates et al., 2015), although the same methods can be applied using other LMM software or to any shared construct(s) measured by multiple‐item scales, which are completed by individual members responding about the group to which they belong. More extensive treatments of LMM are available in various textbooks (e.g., Molenberghs & Verbeke, 1997; Snijders & Bosker, 2012; Hox et al., 2018; Jiang, 2007), and briefer papers demonstrate estimating variance components for G‐coefs with lme4 (Jiang, 2018; Putka et al., 2008; Jorgensen, 2021; Ten Hove et al., 2025).

We interpret point (and interval) estimates because that is the focus of this article, without testing hypotheses about meeting minimal criteria (e.g., Bonett, 2002; Bonett & Wright, 2015; McGraw & Wong, 1996). We used restricted maximum likelihood (REML) estimation because the LMM contains only a fixed intercept and random intercepts associated with components in Equation (5), and variance components of the latter (Equation 7) are our priority for unbiased estimation. Whereas full ML estimation can produce (often downward) biased estimates of variance components by not accounting for the df lost when estimating fixed effects, REML provides more accurate, unbiased variance‐component estimates by ‘partialing out’ the fixed intercept in Equation (5) (Snijders & Bosker, 2012).

We report 90% CIs for all ICCs, obtained using a resampling method often referred to as a Monte Carlo CI (Preacher & Selig, 2012), implemented in the semTools package (version 0.5‐9; Jorgensen et al., 2026). Ten Hove et al. (2025) demonstrated via simulations that Monte Carlo CIs often yield nominal coverage rates when applied to LMMs (see also Jiang et al., 2022, who investigated other resampling methods). A 90% confidence level was chosen for two‐sided CIs, which corresponds to 95% confidence using only the lower bound (e.g., for interested readers to test a $H_{0}$ that reliability exceeds a minimal criterion), but we report both lower and upper limits for completeness.

### 4.1 Practical application

The LMM of interest is represented in Equation (5), which contains a single fixed parameter (the intercept μ), four random intercepts ($\beta_{s}$, $\beta_{i}$, $\beta_{si}$, and $\beta_{t:s}$), and the residual term ($\beta_{(t:s)i,\epsilon }$). The distributional assumptions are that the random intercepts and residuals are independent and normally distributed, with the variance components from Equation (7):

(19) $$\begin{array}{ll} \beta_{s} & ∼N(0,\sigma_{s}^{2}), \end{array}$$

(20) $$\begin{array}{ll} \beta_{i} & ∼N(0,\sigma_{i}^{2}), \end{array}$$

(21) $$\begin{array}{ll} \beta_{si} & ∼N(0,\sigma_{si}^{2}), \end{array}$$

(22) $$\begin{array}{ll} \beta_{t:s} & ∼N(0,\sigma_{t:s}^{2}), \end{array}$$

(23) $$\begin{array}{ll} \beta_{(t:s)i,\epsilon } & ∼N(0,\sigma_{(t:s)i,\epsilon }^{2}). \end{array}$$

We used the R package lme4 (version 1.1.38; Bates et al., 2015) to fit this LMM to the SEAS data provided by Ye et al. (2022). Along with SEM results presented in the next section, the resulting variance‐component estimates are reported in Table 2. Point estimates were plugged into Equations ((9), (11), (14)), and (16) to obtain reliability coefficients reported in Table 3.

**TABLE 2:** Maximum likelihood point estimates of variance components for SEAS data using LMM and SEM.

	LMM	SEM 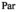	SEM 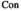
${\hat{\sigma }}_{s}^{2}$	.151	.149	.127
${\hat{\sigma }}_{i}^{2}$	.107	.108	.108
${\hat{\sigma }}_{si}^{2}$	.045	.044	.046
${\hat{\sigma }}_{t:s}^{2}$	.285	.285	.302
${\hat{\sigma }}_{(t:s)i,\epsilon }^{2}$	.295	.295	.282

*Note*: LMM = linear mixed model, using lme4::lmer() to obtain restricted ML estimates. SEM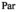 = structural equation model with essentially parallel constraints, using lavaan::cfa() to obtain ML estimates. SEM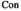 = structural equation model for congeneric items, calculated from ML estimates using weights $w=\frac{1}{4}\;1$ to average over $n_{i}=4$ items.

**TABLE 3:** Point and interval estimates of reliability coefficients for SEAS data using LMM and SEM.

	LMM^a^		SEM 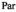 ^b^		SEM 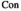 ^b^ ^,^ ^c^	
Coefficient	${\hat{\rho }}^{2}$	90% CI^d^	${\hat{\rho }}^{2}$	90% CI	${\hat{\rho }}^{2}$	90% CI
${\text{ICC}}_{s}(\text{C},n_{t:s}=1,n_{i}=1)$	.194	[.122, .260]	.193	[.122, .258]	—	—
${\text{ICC}}_{s}(\text{C},n_{t:s}=1,n_{i}=4)$	.289	[.186, .380]	.287	[.187, .376]	.249	[.158, .340]
${\text{ICC}}_{s}(\text{C},n_{t:s}={ñ}_{t:s},n_{i}=1)$	.301	[.199, .387]	.300	[.200, .383]	—	—
${\text{ICC}}_{s}(\text{C},n_{t:s}={ñ}_{t:s},n_{i}=4)$	.430	[.298, .532]	.428	[.299, .528]	.381	[.259, .489]
${\text{ICC}}_{s}(\text{A},n_{t:s}={ñ}_{t:s},n_{i}=4)$	.400	[.271, .508]	.397	[.274, .494]	.352	[.237, .455]
${\text{ICC}}_{si}(\text{A},n_{t:s}={ñ}_{t:s},N_{i}=4)\;\;$ ^e^	.462	[.336, .560]	.459	[.336, .556]	.415	[.298, .519]
${\text{ICC}}_{t:s}(\text{C},n_{t:s}={ñ}_{t:s},n_{i}=4)\;\;$ ^f^	.857	[.826, .881]	.857	[.827, .880]	.855	[.825, .880]
${\text{ICC}}_{s}(\text{C},n_{t:s}=7,n_{i}=4)$	.707	[.576, .784]	.705	[.578, .782]	.663	[.528, .753]
${\text{ICC}}_{s}(\text{C},n_{t:s}=14,n_{i}=4)$	.803	[.698, .861]	.803	[.699, .860]	.770	[.654, .840]

*Note*: LMM = linear mixed model, SEM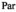 = structural equation model with essentially parallel constraints, SEM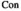 = structural equation model for congeneric items, CI = Monte Carlo confidence interval estimated using semTools::monteCarloCI(), ICC = intraclass correlation coefficient (generically symbolized as $E({\hat{\rho }}^{2})$ for consistency or Φ for agreement in GT literature), ${ñ}_{t:s}=1.908$ is the harmonic‐mean number of teachers per class in the SEAS data.

^a^ Restricted MLE using lme4::lmer().

^b^ MLE using lavaan::cfa().

^c^ Congeneric items are assumed fixed in new samples; thus, ICCs should be reported using a capital $N_{i}=4$ (or $N_{i}^{\text{C}}=4$ to additionally indicate items are congeneric).

^d^ Robust asymptotic covariance matrix estimated using merDeriv::vcov.lmerMod() (Wang & Merkle, 2018).

^e^ Interrater agreement (G‐ or D‐coef given fixed items).

^f^ Inter‐item reliability (G‐coef given fixed raters).

Comparing variance components in Table 2, one can see that there is more school‐level variance ($\sigma_{s}^{2}=0.151$) than item variance ($\sigma_{i}^{2}=0.107$) or their interaction ($\sigma_{si}=0.045$). This is encouraging because the shared SEAS construct’s variance should primarily reflect differences between schools, not differences in how schools differentially respond to items. However, teacher‐level variances are overwhelmingly more influential than school‐level variance, indicating that composites would be quite saturated with measurement error, which seriously undermines comparing schools based on composites of teacher ratings. Indeed, the reliability coefficients in Table 3 can illuminate what conditions would be necessary for such a composite to yield reliable (i.e., generalizable or dependable) data.

Each of the first four rows of Table 3 is a G‐coef (Equation 9) indicating the expected consistency of single or composite scores. The first row of Table 3 indicates that a single teacher's response to a single item of the SEAS parental subscale would not be very generalizable, ${\text{ICC}}_{s}(\text{C},n_{t:s}=1,n_{i}=1)=.194$, 90% CI [.122, .260]—that is, we could not expect the rank‐ordering of schools to be consistent using single‐teacher, single‐item responses. Composite scores are more consistent, by averaging over $n_{i}=4$ items, ${\text{ICC}}_{s}(\text{C},n_{t:s}=1,n_{i}=4)=.289$, 90% CI [.186, .380], or over ${ñ}_{t:s}=1.908$ teachers, ${\text{ICC}}_{s}(\text{C},n_{t:s}=1.908,n_{i}=1)=.301$, 90% CI [.199, .387], but even averaging over both items and teachers, ${\text{ICC}}_{s}(\text{C},n_{t:s}=1.908,n_{i}=4)=.430$, 90% CI [.298, .532], leaves over half of the composite's variance contaminated by measurement error. Because item‐specific error ($\sigma_{i}^{2}=0.107$ in Table 2) is not negligibly smaller than other variance components, absolute agreement (Equation 11) is noticeably lower than consistency, ${\text{ICC}}_{s}(\text{A},n_{t:s}=1.908,n_{i}=4)=.400$, 90% CI [.271, .508], indicating that composite scores calculated from this number of items and teachers per school (and using the mean as a cut score) would not be a reliable (or ‘dependable’) way to classify schools.

One can see in Table 2 that teacher‐related error is the overwhelming majority of variance. Equations 14 and 16 can be used to quantify the (un)reliability of scores due to specific error components. Inter‐item reliability, ${\text{ICC}}_{t:s}(\text{C},n_{t:s}={ñ}_{t:s},n_{i}=4)=.857$, 90% CI [.826, .881], is quite high—even the lower 90% confidence limit exceeds .80—whereas IRR is quite poor, ${\text{ICC}}_{si}(\text{A},n_{t:s}={ñ}_{t:s},N_{i}=4)=.462$, 90% CI [.336, .560], indicating that teacher error is the greater threat to validity of any school‐level inferences drawn from an SEAS composite.

It is well known from CTT that reliability can be improved by adding more scale items, but GT can give researchers a good intuition about why reliability can be improved with larger samples in any facet of generalization. Increasing the number of items ($n_{i}$) or teachers per school ($n_{t:s}$) in Equation (9) reduces the size of those variance components contributing to a composite. The same variance‐component estimates in Table 2 can be plugged into Equation (9) (or 11) with hypothetical sample sizes, to estimate the expected reliability of a composite calculated from more teachers per school. The bottom two rows of Table 3 hold the number of items constant, but increasing $n_{t:s}$ shows that a minimally acceptable reliability of .70 could be achieved with $n_{t:s}=7$ teachers per school. However, using a stricter criterion for minimally acceptable reliability, $n_{t:s}=14$ yields ${\text{ICC}}_{s}(\text{C},n_{t:s}=14,n_{i}=4)=.803$, 90% CI [.698, .861]. Even larger samples would be required for a more conservative approach of using (lower) confidence limits to test whether the population reliability meets a minimum standard (e.g., Bonett, 2002; Bonett & Wright, 2015; McGraw & Wong, 1996), as shown in our OSF project’s R syntax.

## 5 ILLUSTRATION USING SEM

We now present a multilevel CFA, which can be specified analogous^10^ to the LMM in Equation (5). We briefly introduce the multilevel factor model below, using the traditional LISREL notation (Bollen, 1989). We continue using the subscripts associated with our motivating example, but the model description below generalizes to any sort of observations nested within clusters. After briefly discussing similarity of CFA and LMM results, we distinguish conceptual issues about treating items as a fixed or random facet, with consequences for homogeneity assumptions implicit to LMM. We present analogous formulas for the variance components in Equation (7) as functions of (potentially less constrained) CFA parameters, yielding more general definitions of factor‐model‐based reliability coefficients analogous to those in Equations (9), (11), (14), and 16, which allows reliability to be estimated under less‐restrictive measurement conditions than LMM (Vispoel et al., 2021).

### 5.1 Multilevel common factor model

The responses of each teacher in each school to the $n_{i}$ scale items is stored in a vector $y_{t:s}$, which is decomposed into latent components at the school level (i.e., school means; Equation 4) and teacher level (i.e., teacher deviations from their school's mean):

(24) $$y_{t:s}={\tilde{y}}_{s}+(y_{t:s}-{\tilde{y}}_{s}),$$

where the parenthetical term is the within‐school (teacher) component. Each component vector is modelled as a function of f≥1 common factors η at that level of analysis:

(25) $$\begin{array}{llllll} y_{t:s}-{\tilde{y}}_{s} & = & & \Lambda^{\left(\text{W}\right)}\eta_{t:s}^{\left(\text{W}\right)} & + & \varepsilon_{t:s}^{\left(\text{W}\right)}, \end{array}$$

(26) $$\begin{array}{llllll} {\tilde{y}}_{s} & = & \nu + & \Lambda^{\left(\text{B}\right)}\eta_{s}^{\left(\text{B}\right)} & + & \varepsilon_{s}^{\left(\text{B}\right)}, \end{array}$$

where Λ is a $n_{i}\times f$ matrix of factor loadings and ε is a vector of residuals (a ‘unique factor’ per item). Because lower‐level components are mean centered, intercepts ν are only modelled at the highest level of analysis. The common factors are assumed normally distributed at each level of analysis:

(27) $$\begin{array}{ll} \eta_{t:s}^{\left(\text{W}\right)} & ∼\mathrm{MVN}(0,\Psi^{\left(\text{W}\right)}), \end{array}$$

(28) $$\begin{array}{ll} \eta_{s}^{\left(\text{B}\right)} & ∼\mathrm{MVN}(\kappa ,\Psi^{\left(\text{B}\right)}), \end{array}$$

where Ψ is a f×f covariance matrix and κ is a vector of factor means. Likewise, unique factors are assumed normally distributed at each level of analysis:

(29) $$\begin{array}{ll} \varepsilon_{t:s}^{\left(\text{W}\right)} & ∼\mathrm{MVN}(0,\Theta^{\left(\text{W}\right)}), \end{array}$$

(30) $$\begin{array}{ll} \varepsilon_{s}^{\left(\text{B}\right)} & ∼\mathrm{MVN}(0,\Theta^{\left(\text{B}\right)}), \end{array}$$

where Θ is a (typically diagonal) $n_{i}\times n_{i}$ covariance matrix. The model‐implied mean structure of observed variables is:

(31) $$E(y_{t:s})=\nu +\Lambda^{\left(\text{B}\right)}\kappa ,$$

while the model‐implied covariance structure at each level is:

(32) $$\begin{array}{ll} \mathrm{Var}(y_{t:s}-{\tilde{y}}_{s}) & =\Lambda^{\left(\text{W}\right)}\Psi^{\left(\text{W}\right)}\Lambda^{\left(\text{W}\right)}+\Theta^{\left(\text{W}\right)}, \end{array}$$

(33) $$\begin{array}{ll} \mathrm{Var}({\tilde{y}}_{s}) & =\Lambda^{\left(\text{B}\right)}\Psi^{\left(\text{B}\right)}\Lambda^{\left(\text{B}\right)}+\Theta^{\left(\text{B}\right)}. \end{array}$$

When fitting the congeneric CFA in Figure 1 to the SEAS data, we constrain factor loadings to equality across levels ($\Lambda^{\left(\text{W}\right)}=\Lambda^{\left(\text{B}\right)}=\Lambda$), so the school‐ and teacher‐level factors remain interpretable as the GT components indicated in Figure 2 (Jak et al., 2013, 2023; Jak & Jorgensen, 2017). But the formulas we present in the following sections do not strictly require cross‐level^11^ metric invariance.

**FIGURE 2:**
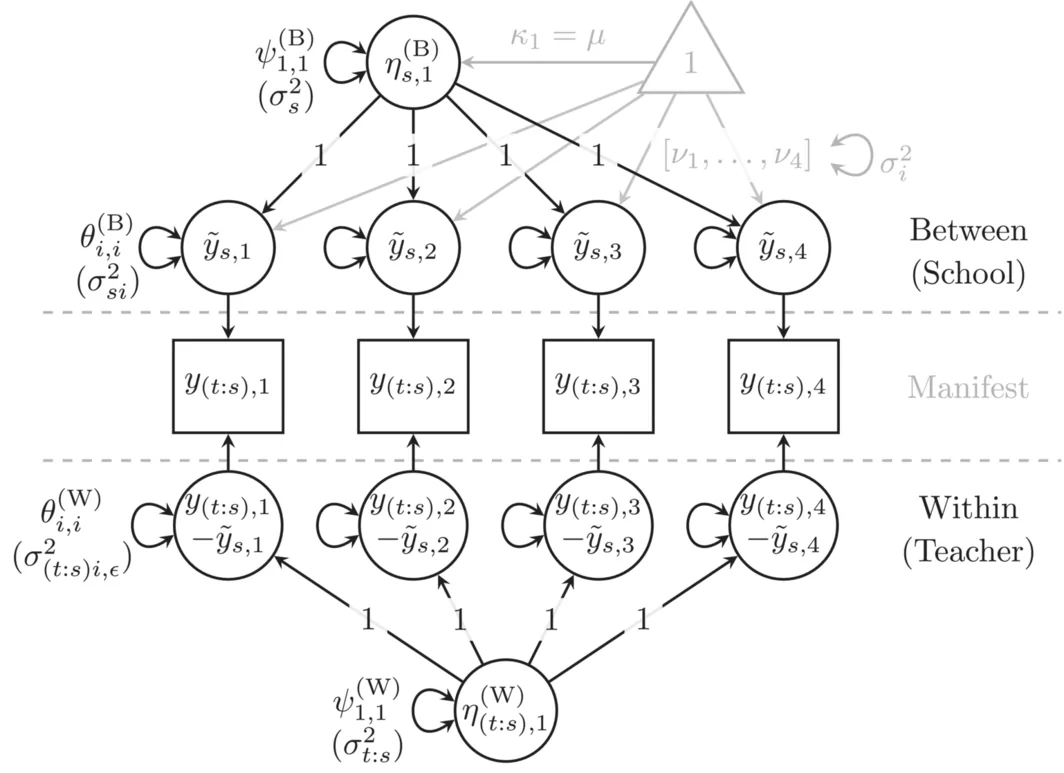
Path diagram depicting a multilevel CFA with the assumption of 4 indicators being essentially parallel items (i.e., equal factor loadings and residual variances across item components) at each level of analysis. Each CFA parameter is labelled using traditional LISREL notation (Bollen, 1989), with analogous GT parameters depicted in parentheses. The unique‐factor variances are constrained to equality, the item intercepts are constrained to average zero to identify the factor mean (grand mean μ from GT), and the variance of intercepts represents the variance component $\sigma_{i}^{2}$ (main effect of items).

The same variance components from Equation (7) can be estimated as variances of common and unique factors in Figure 1, although the absolute‐error component $\sigma_{i}^{2}$ would not be directly estimable. When all factor loadings = 1 (see Figure 2), $\sigma_{i}^{2}$ can be represented as a function of estimated item intercepts:

(34) $${\hat{\sigma }}_{i}^{2}=\frac{1}{n_{i}-1}\sum_{i=1}^{n_{i}}{\hat{\nu }}_{i}^{2},$$

as depicted by the mean structure in Figure 2. The factor mean is estimable by constraining the sum of intercepts to be zero (Jorgensen, 2021), yielding intercepts that are interpretable as item main effects ($\beta_{i}$, i.e., item‐mean deviations around the grand mean κ=μ). Figures 1 and 2 are equivalent when all factor loadings are fixed to 1 and residual variances (at each level of analysis) are constrained to equality across items. The constraints in Figure 2 represent *essentially* 12 *parallel* indicators, reflecting an assumption that items are randomly sampled from a theoretically infinite population of items measuring the same construct (Vispoel et al., 2021).

We used ML estimation in the lavaan package (version 0.7‐2; Rosseel, 2012; Rosseel et al., 2026) to fit the CFA in Figure 2 to the SEAS data provided by Ye et al. (2022). Table 2 shows how similar the variance‐component estimates are between the two modelling frameworks (Jorgensen, 2021; Vispoel et al., 2023), with differences ≤.002 in absolute value across components. Likewise, the reliability coefficients in Table 3 differ ≤.003 in absolute value between those obtained from LMM and SEM estimates. All interpretations and applications are consistent with the LMM reported in the previous section, given the fitted SEM reflects the same essentially parallel assumptions made by LMM. The rest of this section discusses how SEM can be advantageous by relaxing the essentially parallel assumption.

### 5.2 Congeneric items

The multilevel CFA in Figure 2 incorporates essentially parallel assumptions: equality of loadings and residual variances across items. Relaxing the equality constraints on residual variances yields the less strict assumption of essential^13^ *tau‐equivalence*, which only involves equality of factor loadings. The most general CFA in Figure 1 does not impose any equality constraints, conforming to the least restrictive measurement model for *congeneric* items.

The essentially parallel assumption of the previous LMM and CFA applications is easily defensible (and arguably necessary) for the teacher facet, given that teachers constitute a random sample of lower‐level units within the randomly sampled target of measurement (schools). However, items could be considered fixed when the same items are expected to be used for measurements provided in new samples, as we discussed when adapting the G‐coef (Equation 9) to assume fixed items (Equation 14). We view a congeneric model to be conceptually consistent with estimating variance components of fixed facets because fixed‐item parameters are estimated separately rather than assuming item‐related effects are randomly sampled from a common distribution.

Similar to past work (e.g., Brennan, 2001, tables 4.1 & 4.2; McGraw & Wong, 1996, tables 4 & 5), our ICC formulas reflect the same essentially parallel assumption associated with randomly sampled facet levels. Whereas $\sigma_{i}^{2}$ is obtained indirectly as a function of CFA parameters (item means; see Equation 34) regardless of whether items are treated as random or fixed, the remaining components that capture item‐related variance (see distributional assumptions in Equations 21 & 23) would still be estimated by LMM (as well as the CFA in Figure 2) assuming normality and homoskedasticity across levels of the fixed facet. The congeneric CFA in Figure 1 instead relaxes equality assumptions for components $\beta_{si}$ and $\beta_{(t:s)i,\epsilon }$ across items. Because a CFA with essentially parallel constraints is nested under a congeneric CFA, the essentially parallel assumption can be tested^14^ as a $H_{0}$ using a likelihood ratio test.

Calculating reliability coefficients using congeneric CFA parameters requires defining the variance components in Equation (6) in terms of CFA parameters. We provide these definitions in the next section. Furthermore, using a fixed set of items implies only generalizing to situations in which a composite is calculated from those items, in which case the IRR with $N_{i}=4$ in Table 3 should be interpreted. Although we view a congeneric measurement model to be more consistent with treating items as fixed (in which case, no coefficients should include $\sigma_{i}^{2}$), we nevertheless include the G‐ and D‐coefs from Equations 9 and 11 in Table 3. Similar to estimating inter‐item reliability as though nested raters could be fixed, a researcher may also want to consider items as random even when relaxing parallel or tau‐equivalence assumptions.

### 5.3 Variance components implied by CFA parameters

The variance component for schools in Figure 2 is the school‐level factor variance ($\sigma_{s}^{2}=\psi_{1,1}^{\text{(B)}}$). This is actually a simplification of the more general formula for congeneric items, in which the school‐level variance contribution to a composite is a function of both the factor covariance matrix ($\Psi^{\text{(B)}}$) and factor loadings ($\Lambda^{\text{(B)}}$) depicted in Figure 1:

(35) $$\sigma_{s}^{2}=w^{\prime }\Lambda^{\text{(B)}}\Psi^{\text{(B)}}\Lambda^{\text{(B)}}w,$$

where w is a vector of $n_{i}$ weights applied to the items before summing. Optimal weights can be chosen to maximize the composite’s reliability (Li, 1997), but most frequently the (implied) selected weights are a vector of ones (1, yielding a scale sum) or the inverse of the number of summed items ($n_{i}^{-1}1$, yielding a scale mean).

Other variance components constitute measurement error. The variance component for teachers within schools in Figure 2 is the teacher‐level factor variance ($\sigma_{t:s}^{2}=\psi_{1,1}^{\text{(W)}}$). Again, this is a reduction in the more general formula for congeneric items, in which the teacher‐level variance contribution to a composite is a function of both the factor covariance matrix ($\Psi^{\text{(W)}}$) and factor loadings ($\Lambda^{\text{(W)}}$) depicted in Figure 1:

(36) $$\sigma_{t:s}^{2}=w^{\prime }\Lambda^{(\text{W)}}\Psi^{(\text{W)}}\Lambda^{(\text{W)}}w.$$

The variance component for the school × item interaction in Figure 2 is the residual variance of school‐level item components ($\sigma_{si}^{2}=\theta_{i,i}^{\text{(B)}}$), constrained to equality across items. Without requiring equality constraints, the more general formulation takes the weighted sum of the residual covariance matrix:

(37) $$\sigma_{si}^{2}=w^{\prime }\Theta^{(\text{B)}}w,$$

where $\Theta^{\text{(B)}}$ can include residual covariances. Likewise, the variance component for the highest‐order term in Equation (5) is the teacher‐level residual variance ($\sigma_{(t:s)i,\epsilon }^{2}=\theta_{i,i}^{\text{(W)}}$), constrained to equality across items. But the general formulation is less constrained:

(38) $$\sigma_{(t:s)i,\epsilon }^{2}=w^{\prime }\Theta^{\left(\text{W}\right)}w,$$

which accommodates tau‐equivalent or congeneric items.

The final variance component is $\sigma_{i}^{2}$, which is only relevant for estimating absolute error in an agreement/dependability coefficient (Equations 11 or 18). When the factor mean is identified under a sum‐to‐zero constraint on intercepts, their estimates from the parallel model are interpretable as item main effects $\beta_{i}$ in Equation (5) (Jorgensen, 2021); however, model‐implied means $\hat{\mu }$ from the congeneric model are functions not only of the intercepts and factor mean, but also the factor loading (i.e., Equation 31). Instead, we can estimate $\sigma_{i}^{2}$ as a sum of squared (potentially weighted) deviations of model‐implied item means from the model‐implied grand mean (i.e., the mean of means: $\bar{\mu }=\frac{1}{n_{i}}\sum_{i=1}^{n_{i}}{\hat{\mu }}_{i}$):

(39) $$\sigma_{i}^{2}=\frac{1}{n_{i}-1}\sum_{i=1}^{n_{i}}{\left[w_{i}({\hat{\mu }}_{i}-\bar{\mu })\right]}^{2}.$$

Note that Equation (39) is equivalent to Equation (34) when using unit weights and the intercepts are already deviations from the grand mean.

### 5.4 Reliability for fixed congeneric items

Calculating reliability coefficients from estimated factor‐model parameters has a long history (Bentler, 1968; Heise & Bohrnstedt, 1970; Jöreskog, 1971; McDonald, 1970). Although these coefficients have often been denoted ω (Cho, 2021) to contrast with coefficient α (Cronbach, 1951), Cho (2016) argued for a more systematic naming convention that denotes reliability estimates ($\hat{\rho }$) with subscripts that indicate whether estimation assumes parallel (${\hat{\rho }}_{\text{P}}$), tau‐equivalent^15^ (${\hat{\rho }}_{\text{T}}=\alpha$), or congeneric items (${\hat{\rho }}_{\text{C}}=\omega$). Our suggested ICC notation builds on conventions provided by McGraw and Wong (1996), whose notation did not distinguish between (a) fixed and random facets, nor between (b) parallel, tau‐equivalent, or congeneric assumptions. Doing so can quickly become complicated in designs with more than one facet of generalization, as these distinctions can apply independently to different facets—for example, our continuing example below will treat items as fixed and congeneric, but teachers as random and parallel.

In this paper, we follow the convention of using a capital N to represent a fixed‐facet's sample size, and we advocate treating fixed facets as congeneric in order to relax distributional assumptions. In such a case, the capital N could simultaneously imply the facet is fixed and congeneric. However, we recognize that researchers may want to treat items as fixed while fitting a model that assumes they are essentially parallel, especially if they employ software that does not permit relaxing the equality constraints necessary to reflect congeneric measurement. We do not attempt to resolve this notational distinction here, but the subscripts proposed by Cho (2016) could be applied as superscripts to each facet's sample size (e.g., $N_{i}^{\text{C}}=4$).

### 5.5 Practical application

We used lavaan::cfa() to fit the CFA in Figure 1 (with metric‐invariance constraints; Jak & Jorgensen, 2017; Jak et al., 2023) to the SEAS data, reporting results in the rightmost columns of Tables 2 and 3. The congeneric estimates in Table 2 are calculated using weights $w=\frac{1}{N_{i}}\;1$, so the composite is a mean across items, thus on the same scale as the items. Variance components in our R syntax on OSF are scaled to make their estimates from the congeneric model comparable to those from the essentially parallel model, but this scaling is not generally necessary to calculate ICCs because the scaling weights in the numerator and denominator cancel out.

The G‐coefs in the first four rows of Table 3 continue to indicate unacceptably low reliability, although congeneric reliability estimates (e.g., .38 when averaging across teachers and items) are notably lower than corresponding parallel reliability estimates (i.e., .43). This seems to be driven largely by less school variance yet more teacher‐level error, as inter‐item reliability only differs in the third decimal (.855 vs. .857), whereas IRR (which is also a G‐coef assuming fixed items) is lower for congeneric (.415) than parallel (.46) ICCs. The bottom two rows of Table 3 indicate the number of teachers per school necessary for parallel‐model estimates to meet common reliability benchmarks (ρ>.70 or .80). The congeneric coefficients are lower, failing to meet these benchmarks.

## 6 CONNECTIONS WITH PAST RESEARCH

For the context under consideration in our paper (shared constructs measured by cluster members' item responses), Brennan (2001, equations 4.31–4.33) originally proposed ICCs equivalent to our Equations (9), (16), and 14 to reflect generalizability of scores across cluster members, items, or both. He interpreted the coefficients that generalize over only one of the two facets of generalization (which we label as inter‐item reliability and IRR) as treating the other facet as fixed, which coincides with interpretations of our coefficients as conditional correlations (Lin et al., 2025; Vangeneugden et al., 2005). In our view, the inter‐item reliability and IRR coefficients have practical utility even if both facets of generalization are considered random, insofar as they can provide more information (i.e., beyond merely considering different sample sizes in a decision study) about which sources of error pose greater risks to valid comparisons between schools, or to comparing a single school to a given criterion.

Several related reliability coefficients and ICCs have been proposed for analogous multilevel designs. Ten Hove et al. (2022, Design 1) proposed IRR coefficients for designs in which raters observe targets who are nested in clusters, and measurements are made using a single item (see also Lin et al., 2025, who proposed different coefficients for Design 1). Our paper focusses on designs in which the raters are the Level‐1 units, the group they are nested in is the target, and measurements are made using multiple items. In this section, we review existing multilevel reliability coefficients that can fall within that scope. Table 1 shows how and under what conditions they are related or equivalent to coefficients defined in Equations (9), (11), (14), (16), and 18 for given choices of $n_{i}$ and $n_{t:s}$, and we provide additional formulas and point/interval estimates where necessary (e.g., when $n_{i}$ or $n_{t:s}$ differ from Table 3). ICCs estimates in the following section that are excluded from Table 3 are available from our supplementary materials on OSF.

### 6.1 Coefficients for a single multilevel variable

Textbooks on LMM (or multilevel modelling) often begin with the so‐called empty or intercept‐only model, in order to estimate an outcome variable's ICC (Hox et al., 2018, equation 2.9; Singer & Willett, 2003, equation 4.7; Snijders & Bosker, 2012, equations 3.2, 3.19, and 4.7). In univariate 2‐level models, this ICC represents the proportion of variance in the outcome that is attributable to differences between clusters, and so can be explained by Level‐2 covariates. Notation varies across textbooks, but the format always matches Equation (8) with $\sigma_{T}^{2}$ replaced by the variance of Level‐2 means (i.e., between‐level variance: $\sigma_{B}^{2}$) and $\sigma_{E}^{2}$ replaced by the remaining variance within Level‐2 units ($\sigma_{W}^{2}$):

(40) $${\text{ICC}}_{\text{O}}=\frac{\sigma_{\text{B}}^{2}}{\sigma_{\text{B}}^{2}+\sigma_{\text{W}}^{2}}.$$

Bliese (2000) referred to this as ICC(1), which should not be confused with the reliability coefficient that McGraw and Wong (1996) also coined ICC(1), which we discuss below. We use the ‘O’ subscript for ‘observed’, following Kush et al. (2022, equation 11), who distinguished ICCs for observed and latent variables in multilevel SEM. We discuss the latter in an upcoming section.

When the outcome variable is meant to measure a cluster‐level construct (as in our motivating example), the cluster members can be seen as ‘raters’ of their cluster. In such a case, Equation (40) can be interpreted as an IRR coefficient, which Shrout & Fleiss (1979, p. 423) referred to as ICC(1,1) and McGraw & Wong (1996, table 4) referred to as ICC(1). Both of these express the reliability of a single rater’s scores on a single indicator of the construct—in our motivating example, the reliability of scores from one teacher per school, using only a single item to measure parental SEAS. Table 1 lists this coefficient in the same section as the earlier IRR and inter‐item coefficients because they reflect reliability of observed (or observable future) variables, although in this case it is only a single variable.

The univariate IRR coefficient in Equation (40) is related to the IRR coefficient for a (t:s)×i design in Equation (14). When only one scale item is used to measure the parental SEAS construct, between‐item differences cannot be modelled in Equation (5), so the variance component $\sigma_{si}^{2}$ becomes confounded with $\sigma_{s}^{2}$, and $\sigma_{(t:s)i,\epsilon }^{2}$ becomes confounded with $\sigma_{t:s}^{2}$. Therefore, those variance components disappear from Equation (14) when using only $n_{i}=1$ item, reducing ${\text{ICC}}_{si}(\text{C},n_{t:s}=1,n_{i}=1)$ to:

(41) $$\text{ICC}(1)=\frac{\sigma_{s}^{2}}{\sigma_{s}^{2}+\sigma_{t:s}^{2}},$$

which superficially appears equivalent to ICC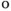 in Equation (40), although the confounding makes its value equivalent to ${\text{ICC}}_{si}$. The absence of item‐specific variance components from this calculation does not indicate that the single‐indicator measurements lack item‐specific error; rather, a single‐indicator design does not allow item‐specific error to be disentangled from other sources of variance.

Across the four items in our example, the range of ICC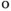 estimates was .198–.295, the mean of which (M= .249) is quite close to the expected value implied by our estimated LMM parameters—${\text{ICC}}_{si}(\text{C},n_{t:s}=1,n_{i}=1)=.252$, 90% CI [.181, .317]—and by the SEM with parallel constraints: ${\text{ICC}}_{si}(\text{C},n_{t:s}=1,n_{i}=1)=.249$, 90% CI [.181, .313]. Model‐implied ICC(1) per item can also be calculated from the congeneric SEM estimates, as functions of item‐specific CFA parameters:

(42) $$\text{ICC(1) for Item}\;i=\frac{\lambda_{i,1}^{\text{(B)}}\psi_{1,1}^{\text{(B)}}\lambda_{i,1}^{\text{(B)}}+\theta_{i,i}^{\text{(B)}}}{\lambda_{i,1}^{\text{(B)}}(\psi_{1,1}^{\text{(B)}}+\psi_{1,1}^{\text{(W)}})\lambda_{i,1}^{\text{(B)}}+\theta_{i,i}^{\text{(B)}}+\theta_{i,i}^{\text{(W)}}}.$$

We imposed metric‐invariance constraints in our congeneric SEM, so $\lambda_{i,1}^{\text{(B)}}=\lambda_{i,1}^{\text{(W)}}$. These item‐specific IRR estimates had a smaller range (.223–.238, M=.230) than the models that assumed parallel indicators.

### 6.2 Coefficients for latent components

Hypothetically, one could imagine an indicator that does not contain any item‐specific measurement error, or imagine being able to directly observe the latent multilevel variable η in Figures 1 and 2 and Equations 27 and 28:

(43) $$\eta_{t:s}=\eta_{t:s}^{\left(\text{W}\right)}+\eta_{s}^{\left(\text{B}\right)}.$$

The same coefficient in Equations 40 and 41 could then hypothetically be calculated for $\eta_{t:s}$, indicating the (hypothetical) IRR for measurements free of item‐specific error. Despite the latent variable $\eta_{t:s}$ not being observable (hence shown in the lowest section of Table 1), one can calculate this ‘hypothetical reliability’ as a function of the variance components for $\eta_{t:s}^{\left(\text{W}\right)}$ and $\eta_{s}^{\left(\text{B}\right)}$:

(44) $${\text{ICC}}_{\text{L}}=\frac{\psi_{1,1}^{\text{(B)}}}{\psi_{1,1}^{\text{(B)}}+\psi_{1,1}^{\text{(W)}}}.$$

Analogous to ICC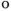, Kush et al. (2022, equation 14) labelled this ICC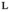 to indicate it would be the latent variable’s ICC. For configural constructs (i.e., representing characteristics of Level‐1 units; Stapleton et al., 2016; Jak et al., 2023), ICC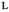 is useful to interpret as the proportion of individual differences in the construct that can be attributed to differences between clusters. For a shared construct (e.g., our parental SEAS example), it can be interpreted analogous to the IRR coefficient ICC(1), if raters were able to directly observe the latent group‐level construct.

Note that interpreting $\eta_{s}^{\left(\text{B}\right)}$ as the cluster‐level means of $\eta_{t:s}$ implies metric invariance (equality of factor loadings) across clusters, which further implies equivalent factor loadings across levels (Jak et al., 2013; Jak & Jorgensen, 2017). In our supplemental materials on OSF, the congeneric SEM we fitted to obtain estimates in Table 2 reflected this metric‐invariance assumption. Furthermore, the latent scale can be identified by estimating both the within‐ and between‐level factor variance under the constraint that their sum is 1, in which case the estimated ${\hat{\psi }}_{1,1}^{\text{(B)}}=.296$, 95% CI [.171, .422], is already a direct estimate of ICC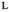. By comparison, the SEM with parallel constraints yielded a higher estimated ICC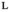 = .343, 90% CI [.234, .452], consistent with parallel vs. congeneric coefficients in Table 3.

Geldhof et al. (2014) also proposed reliability coefficients for latent components of multilevel variables, but rather than IRR coefficients^16^, they proposed (hypothetical) inter‐item reliability coefficients for configural constructs. They proposed applying single‐level composite‐reliability formulas (α or model‐based ω) to the latent components at each level of analysis (right‐hand side of Equation 24). Level‐2 model‐based reliability would thus be calculated only from measurement parameters in Equations (26), (28), and 30, omitting all within‐level variance that reflect differences between observations within a given cluster. In terms of the inter‐item reliability coefficient we proposed in Equation (16) for our motivating example, the between‐level coefficient proposed by Geldhof et al. (2014, see also Lai, 2021, equation 7) omits variance among teachers within the same school:

(45) $$\frac{\sigma_{s}^{2}}{\sigma_{s}^{2}+\frac{\sigma_{si}^{2}}{n_{i}}}.$$

For our example data, Geldhof et al.'s Level‐2 $\hat{\alpha }=.928$ and $\hat{\omega }=.917$ both indicate very little item‐specific error among school averages. This is a common finding, consistent with only minor violations of scalar invariance across clusters (i.e., differences in intercepts across clusters are captured by nonzero residual variances of indicators' Level‐2 components; Jak & Jorgensen, 2017).

Compared to the ICC
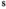
(C, $n_{t:s}={ñ}_{t:s},n_{i}=4$) estimates in Table 2, Geldhof et al.'s (2014) high Level‐2 ‘reliability’ coefficients have a dubious interpretation. Instead of measurements that are free of item‐specific error, imagine measurements that are free of rater error, such that any teacher within the same school would provide the same score (using a particular item) as any other teacher from the same school. One might then interpret the cluster‐level construct as ‘global’ rather than “shared” (Jak et al., 2023). Granted, the multilevel CFA does not assume this because within‐level variance is modelled. However, the coefficient in Equation (45) reflects the hypothetical reliability of a composite calculated from each item’s latent cluster mean ($\tilde{y}$), which is not a composite one could calculate in practice, and greatly exaggerates how reliable an actual Level‐2 composite would be (Lai, 2021). Because it is not a true reliability coefficient (i.e., it does not reflect reliability of an observed or at least observable variable), it is also included in the bottom section of Table 1.

### 6.3 Coefficients for a cluster composite

Relative to sampling a single teacher's rating per school, cluster means are more reliable estimates of a cluster‐level construct, as the error variance per school is proportionally minimized relative to the target variance present in each teacher's rating. The reliability of cluster means is expressed by dividing $\sigma_{\text{W}}^{2}$ in Equation (40) (or $\sigma_{t:s}^{2}$ in Equation 41) by the number of Level‐1 units per cluster (e.g., $n_{t:s}$ in our motivating example):

(46) $$\text{ICC}(n_{t:s})=\frac{\sigma_{\text{B}}^{2}}{\sigma_{\text{B}}^{2}+\frac{\sigma_{\text{W}}^{2}}{n_{t:s}}}=\frac{\sigma_{s}^{2}}{\sigma_{s}^{2}+\frac{\sigma_{t:s}^{2}}{n_{t:s}}}=\frac{n_{t:s}\times {\text{ICC}}_{\text{O}}}{1+(n_{t:s}-1){\text{ICC}}_{\text{O}}}.$$

Bliese (2000) referred to this as ICC(2), regardless of the cluster size, which can equivalently be expressed as a function of ICC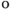 and the (average) cluster size, as in the far‐right term of Equation (46) (see also Snijders & Bosker, 2012, equation 3.21). Shrout and Fleiss (1979) referred to Equation (46) as ICC(1,k) and McGraw & Wong (1996, table 5) referred to it as ICC(k), where k is the number of raters. For unbalanced cluster sizes (as in our motivating example), the harmonic‐mean cluster size is used ($k={ñ}_{t:s}=1.908$; Putka et al., 2008).

Across the four items in our example, the range of ICC(1.908) estimates was .320–.445, the mean of which (M=.387) is quite close to our estimated ${\text{ICC}}_{si}(\text{C},n_{t:s}=1.908,n_{i}=1)=.391$, 90% CI [.300, .468], obtained from LMM parameters, as well as from the SEM with parallel constraints: ${\text{ICC}}_{si}(\text{C},n_{t:s}=1,n_{i}=1)=.388$, 90% CI [.296, .465]. Model‐implied ICC(1.908) per item was also calculated from the congeneric SEM estimates, dividing the within‐level variances ($\psi_{1,1}^{\text{(W)}}$ and $\theta_{i,i}^{\text{(W)}}$) by 1.908 in Equation (42). As with ICC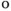 = ICC(1), these item‐specific ICC(1.908) estimates had a smaller range (.354–.373, M=.363) than the models that assumed parallel indicators.

### 6.4 Coefficients for a composite of items

Lai (2021) proposed some valid reliability formulas for observable composites calculated from multilevel, multiple‐item scales, some of which are equivalent to ones we proposed here specifically for cluster‐level constructs. Lai (2021) criticized^17^ Geldhof et al. (2014) for applying composite‐reliability formulas to a situation where a composite cannot actually be calculated. Although Geldhof et al. (2014, p. 75) referred to GT as a guideline for variance decomposition, they did not propose a G‐coef for a composite at either level of analysis. Lai (2021) did not refer to GT, making reference only to CTT; however, he defined some reliability coefficients that are consistent with G‐coefs, despite the CTT framework not allowing for multiple sources of error. The lack of connection to GT in these two papers might follow from mistakenly assuming that GT requires the parallel assumption, given Vispoel et al. (2021) had not yet published their paper showing how GT can incorporate congeneric measurement models via SEM (see also Vispoel et al., 2022).

When calculating a composite to represent a shared construct, by averaging over both raters and items, we propose the G‐coef in Equation 9 (see also Brennan, 2001, equation 4.31), reported as ${\text{ICC}}_{s}(\text{C},n_{t:s}=1.908,n_{i}=4)$ in Table 3. Our G‐coef is equivalent^18^ to what Lai (2021, equations 24 & 17 for $\alpha^{b}$ & $\omega^{b}$, respectively) proposed for a Level‐2 composite of a configural construct (see Table 1). For a shared construct, Lai (2021, equation 20) proposed a similar formula that replaces within‐level CFA parameters with a saturated within‐level covariance matrix. This follows from Stapleton et al. (2016) proposing that measurement of shared constructs should be modelled without imposing constraints, which is a recommendation we criticize in an upcoming section.

Regarding the same composite (i.e., averaging over both raters and items to represent a shared construct), we proposed a inter‐item reliability coefficient in Equation (16) to represent the proportion of composite‐score variance not associated with item‐specific measurement error, which is reported in Table 3 as ${\text{ICC}}_{t:s}(\text{C},n_{t:s}={ñ}_{t:s}=1.908,n_{i}=4)$. One could also be interested in an inter‐item reliability coefficient if a single rater per cluster were sampled, so the composite only averages over items. We would label this ${\text{ICC}}_{t:s}(\text{C},n_{t:s}=1,n_{i}=4)$, which is equivalent to what Lai (2021, equations 23 and 13 for $\alpha^{2l}$ and $\omega^{2l}$, respectively) (see Table 1) proposed for an ‘overall’ composite of a configural construct (i.e., to represent Level‐1 scores without cluster‐mean centering to decompose the Level‐1 and ‐2 components). From the SEM with parallel constraints, the estimated ${\text{ICC}}_{t:s}(\text{C},n_{t:s}=1,n_{i}=4)=.837$, 90% CI [.811, .858], is quite similar to the estimate from the congeneric SEM, ${\text{ICC}}_{t:s}(\text{C},n_{t:s}=1,n_{i}=4)=.839$, 90% CI [.814, .860], which is consistent with inter‐item reliabilities being quite similar in Table 3.

Note that the ${\text{ICC}}_{t:s}(\text{C},{ñ}_{t:s}=1,n_{i}=4)$ estimates above are asymptotically equivalent to simply applying a inter‐item reliability formula for single‐level data to the sample of $N_{t}$ teachers, ignoring that they are nested in schools. Geldhof et al. (2014) advised against doing so, expressing concern that this estimate of reliability would be biased if reliability differed across levels—a hypothesis they confirmed when simulating data from a model in which the metric‐invariance assumption did not hold. However, their conception of level‐specific reliability presupposes that the construct exists independently at each level of analysis, whereas we view the measurement of a shared construct as necessitating the metric‐invariance assumption^19^ by definition (Jak et al., 2023). From the GT perspective, ignoring the nesting of teachers in schools reduces the design to t×i, leaving variance components confounded (see our discussion of IRR in Equation 41). This reduces ${\text{ICC}}_{t:s}(\text{C},n_{t:s},n_{i})$ in Equation (16) to

(47) $${\text{ICC}}_{t}(\text{C},n_{i})=\frac{\sigma_{t}^{2}}{\sigma_{t}^{2}+\frac{\sigma_{ti}^{2}}{n_{i}}},$$

where $\sigma_{s}^{2}$ and $\sigma_{t:s}^{2}$ are confounded in $\sigma_{t}^{2}$, and $\sigma_{si}^{2}$ and $\sigma_{(t:s)i,\epsilon }^{2}$ are confounded in $\sigma_{ti}^{2}$. In the population, this leads to the same inter‐item reliability coefficient one would obtain by modelling the multilevel structure and including those (unconfounded) variance components in Equation (16). In finite samples, however, the unconfounded variance components in ML‐SEM can vary independently (leading to differences in sample estimates) and will generally be estimated with less precision because a ML‐SEM is more highly parameterized than a single‐level SEM, which estimates fewer (but confounded) variance components. In Table 3, the ${\text{ICC}}_{t:s}(\text{C},{ñ}_{t:s}=1,n_{i}=4)=.855$, 90% CI [.825, .879], CI width = .054, estimated from ML‐SEM is slightly higher than a inter‐item reliability coefficient estimated from a single‐level SEM, ICC
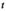
(C, $n_{i}=4$) = .842, which has a narrower 90% CI [.820, .861], CI width = .041.

### 6.5 Coefficients from a shared‐and‐configural model

In his appendix, Lai (2021, equation A1) also proposed how to calculate a reliability coefficient for a shared‐construct composite ($\omega^{b(s)}$), using estimates from the ‘shared‐and‐configural’ model proposed by Stapleton et al. (2016, see also Stapleton & Johnson, 2019). Some conceptual and methodological problems of this model were discussed by Jak et al. (2023), most of which are only indirectly relevant to defining reliability coefficients for shared constructs (e.g., insofar as they reveal threats to the model’s validity). Here, we discuss limitations of the shared‐and‐configural model (depicted in Figure 3) that become apparent from the GT perspective, casting doubt on the usefulness of a reliability coefficient calculated from that model.

**FIGURE 3:**
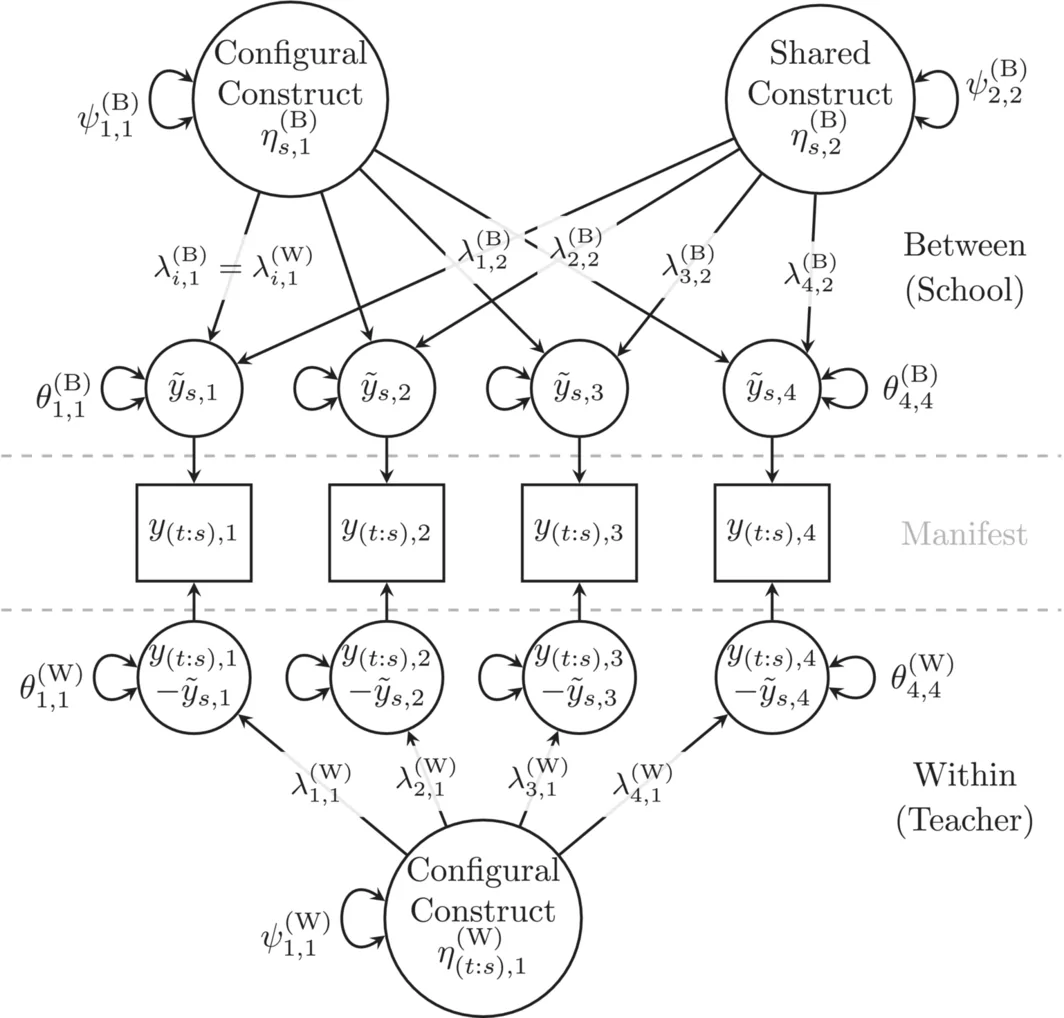
Path diagram depicting a 4‐indicator multilevel CFA with both shared and configural factors, as proposed by Stapleton et al. (2016) and advocated by Stapleton and Johnson (2019).

#### 6.5.1 Substantive interpretation

The GT model in Equation (5) includes a random effect of schools ($\beta_{s}$), which is analogous to CTT's true scores for a shared construct measured with this (t:s)×i design. This effect is represented by the between‐level common factor ($\eta_{s,1}^{\text{(B)}}$) in Figures 1 and 2. The shared‐and‐configural model includes two such random effects, represented as two orthogonal common factors in the Level‐2 model of Figure 3. This Level‐2 factor structure resembles an exploratory factor analysis (EFA) model, with the restriction that the loadings of one factor (i.e., labelled the ‘configural’ factor) are equated with the within‐level factor loadings. Stapleton and Johnson (2019) argued that the configural factor reflects individual perceptions of the shared construct ($\eta_{s,1}^{\text{(W)}}$), rather than the actual shared construct itself, yet Stapleton and Johnson (2019) argue it still has a cluster‐level component ($\eta_{s,1}^{\text{(B)}}$) that is independent from the shared construct (now represented by $\eta_{s,2}^{\text{(B)}}$).

When a shared construct is measured by group members, we agree that measurements reflect the raters’ individual perceptions—otherwise, it would be a global rather than shared construct (Jak et al., 2023). Controlling for item‐specific differences, the decomposition in Equation (5) has terms for what is shared among individual perceptions ($\beta_{s}$) as well as how those perceptions differ among individuals rating the same cluster ($\beta_{t:s}$). Neither CTT nor GT require interpreting $\beta_{s}=\eta_{s}^{\text{(B)}}$ as strictly reflecting the target construct; rather, a true (or universe) score is the average across hypothetically or actually repeated measurements (e.g., Raykov & Marcoulides, 2011, p. 118). Accordingly, reliability (or generalizability) coefficients reflect the squared correlation of individual scores with true (or universe) scores, not necessarily with scores on the actual construct of interest.

Naturally, it is often of theoretical interest to determine the proportion of variance that is not merely reliable, but also valid (i.e., attributable to the target construct). Bifactor modelling, for example, allows for computing not only a reliability coefficient (so‐called ‘total’ $\omega_{\text{T}}$) but also so‐called “hierarchical” $\omega_{\text{H}}$ (McDonald, 1999; Zinbarg et al., 2006), which is the proportion of variance due to the target construct, omitting any reliable but nuisance variance of composite scores attributable to ‘method’ factors. Stapleton and Johnson (2019) suggested that the shared‐and‐configural model could disentangle ‘actual, objective safety of the neighbourhood (a shared cluster‐level characteristic) and the individual’s tolerance for unsafe conditions’ (p. 314), but doing so would require a measurement design that allows multiple sources of (co)variance to be disentangled. Their illustrative example also analysed TIMSS data: a teacher‐level construct *engaging teaching*, measured by multiple students per teacher with $n_{i}=6$ items. Stapleton and Johnson (2019) suggested the within‐level factor could represent raters' *acquiescence*, though that is just one among ‘a range of possible nuisance characteristics or conditions’ (p. 320). A long methodological history of modelling acquiescence bias would suggest that an acquiescence factor should load on items across more than one construct, with loadings constrained to equality (similar to parallel constraints in Figure 2; Billiet & McClendon, 2000; Maydeu‐Olivares & Coffman, 2006; Savalei & Falk, 2014). Although we acknowledge that acquiescence is a theoretically justifiable source of variance to expect in students’ responses to the TIMSS indicators of engaging teaching, we would caution against concluding that a configural factor $\eta_{s,1}^{\text{(B)}}$ (distinct from the shared construct $\eta_{s,2}^{\text{(B)}}$ in Figure 3) has captured that (or any specific source of) variance. In fact, given the propensity of a secondary factor (as in EFA) to capture any source of multidimensionality, nonlinearity, etc., we are more inclined to interpret Stapleton and Johnson's (2019) ‘configural’ factor as representing the target construct and the factor they labelled ‘shared’ as representing nuisance variance.

#### 6.5.2 Identifiability

Taking the GT model in Equation (5) as a starting point, the two between‐level factors of the shared‐and‐configural model would be analogous to having two random intercepts for schools:

(48) $$y_{(t:s)i}=\mu +(\gamma_{s}+\delta_{s})+\beta_{i}+\beta_{si}+\beta_{t:s}+\beta_{(t:s)i,\epsilon },$$

where $\gamma_{s}$ and $\delta_{s}$ represent the shared and configural factors, respectively. Equation (48) reveals a practical limitation that the model would be underidentified, as the random effects $\gamma_{s}$ and $\delta_{s}$ would be perfectly confounded. The congeneric model allows the shared and configural factors to be distinguished only in the case that one factor’s pattern of loadings is not proportionally equivalent to the other factor's pattern. It may be unlikely in practice that two distinct factors have exactly proportional patterns of factor loadings^20^, but even approximately proportional patterns could yield predictable convergence limitations observed by Stapleton and Johnson (2019). For example, if the configural construct in Figure 3 had loadings ${\left[.8,.8,.6,.4\right]}^{\prime }$ and the shared construct had loadings ${\left[.5,.5,.4,.3\right]}^{\prime }$, the two factors might not be empirically distinguishable.

For Stapleton and Johnson (2019) to be able to estimate the shared‐and‐configural model parameters, they imposed certain identifiability conditions that we think render their reported solution(s) suspect. They fixed the first indicator's factor loading of each factor to 1, estimating all 3 factor variances,^21^ but under the constraint that the configural factor's ICC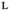 had a fixed value. In fact, their model would not converge unless they fixed ${\text{ICC}}_{\text{L}}<.15$ (Stapleton et al., 2016, p. 323), which we suspect is a sign that $\eta_{s,1}^{\text{(B)}}$ already captured the shared‐construct variance, rendering the secondary $\eta_{s,2}^{\text{(B)}}$ factor redundant. This would be hidden by artificially limiting the degree to which $\eta_{s,1}^{\text{(B)}}$ could contribute shared variance to the indicators, thus allowing for $\eta_{s,2}^{\text{(B)}}$ to contribute the ‘missing’ between‐level (co)variance.

Stapleton and Johnson (2019) did acknowledge the arbitrariness of fixing ICC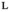 to unknown values, suggesting that ‘additional items, measured at the cluster level’ (p. 325) could be used to identify a model, but the existence of such an item (i.e., not determined by individual perceptions) might render individual‐level responses unnecessary. Perhaps if directly obtaining such cluster‐level measurements were ‘expensive’, then collecting it for a subset of clusters could allow valid construct variance to be distinguished from aggregated individual perceptions, analogous to a two‐method planned missing‐data design (Graham et al., 2006).

## 7 DISCUSSION

This paper used GT to propose a taxonomy of ICCs (summarized by Table 1) that quantify composite reliability of cluster‐level constructs measured by cluster members’ responses to multiple items, the latter of which can be treated as random parallel measurements or as fixed, congeneric items with uniquely estimable parameters. We demonstrated how to estimate variance components and ICCs using open‐source R packages for LMM and SEM, with data and R scripts available on the OSF. We showed that many previously proposed reliability coefficients for this context fit within our taxonomy, criticising the validity of some (e.g., Geldhof et al., 2014; Ten Hove et al., 2022) yet defending the validity of others (e.g., inter‐item reliability of a cluster composite, ignoring the clustering of individual responses) on the condition they are interpreted correctly.

### 7.1 Advice

Researchers should clearly communicate their measurement design, which informs which ICC nomenclature to use when reporting reliability estimates. For example, residents nested in neighbourhoods (r:n) might rate their neighbourhood safety (a group‐level target construct) with multiple items (i)—this could be described as a (r:n)×i design, for which the G‐coef in Equation (9) could be labelled ${\text{ICC}}_{n}(\text{C},n_{r:n},n_{i})$.

Various ICCs can be reported, depending on the context to which it is meant to apply. For example, if a researcher is using the current sample to estimate reliability of data they plan to obtain in the future, then varying $n_{i}$ and $n_{t:s}$ in Equations (9), (11), (14), (16), and 18 can inform how the design protocol could balance costs associated with more items or individuals in order to obtain more reliable composites. However, researchers often use the same data to estimate reliability and to test substantive hypotheses about how the measured construct relates to other variables. In this case, ICCs should be calculated using $n_{i}$ and $n_{t:s}$ that reflect the actual design.

Researchers should bear in mind that only the G‐coef in Equation (9) reflects the degree to which estimated correlation/regression coefficients would be attenuated by all sources of measurement error accounted for by the measurement design. The IRR and inter‐item reliability coefficients can inform which design features introduce more error than others, but they do not necessarily reflect the actual reliability of the composite, unless one of the facets is expected to be fixed (i.e., identical in future samples). For the same reason, we do not generally recommend (hypothetical) reliability coefficients for latent components (e.g., ICC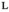 or Geldhof et al.'s (2014) level‐specific coefficients, in the bottom rows of Table 1) because they lack the key characteristic of reflecting the degree^22^ of an actual composite variable's attenuated effect.

Researchers often report reliability estimates from previous research. We recommend providing both point and interval estimates for any reported ICCs, to transparently reflect uncertainty about the true reliability. Because specific goals may not always match those of previous research, researchers should strive to provide as much information as possible to enable readers to calculate any particular ICC of interest. Thus, we recommend that researchers always report all variance‐component estimates from a fitted LMM—or all parameters from a multilevel CFA—so that readers can calculate point estimates of any ICCs of interest for their particular goals. For readers to also obtain interval estimates for their ICCs, it would be necessary to share the raw data (preferably with software scripts), which we encourage whenever it would not risk the privacy of participants who provided their data.

### 7.2 Limitations and future directions

For unbalanced designs, the harmonic mean (ñ, Equation 13) can be used as the sample size for that component in ICC formulas (Putka et al., 2008). This is a general solution for incomplete data (Ten Hove et al., 2024), enabling ICC estimation with planned missing data. For example, using the three‐form design (Graham et al., 1996) with our motivating example, each teacher would have responded to only 3 out of 4 indicators. Thus, this 4‐item design would yield composites calculated with $n_{i}=3$ indicators, so $n_{i}=3$ should be used in reliability formulas.

This is straightforward with parallel items, but congeneric items have varying reliability. Thus, reliability will differ between subsamples of participants who completed the three different forms. In this case, it may be preferable to calculate reliability for each composite, by applying Equations (35), (36), (37), (38), (39) with weights $w_{i}=0$ for items excluded from a composite. When there are additional unplanned missing values, the harmonic mean ñ could be used within each subsample. However, future research may reveal how to calculate an overall reliability coefficient for composites calculated from varying subsets of congeneric items.

## 8 CONCLUSION

GT provides a flexible framework for ICCs to be defined for complex measurement designs, expressing reliability across various dimensions of measurement error. When measuring group‐level constructs with responses of group members to multiple‐item scales, ICCs can be reported to express inter‐item reliability, IRR, or generalizability across both items and raters. This allows researchers to evaluate measurement procedures in fine‐grain detail and predict the most efficient way to improve the measurement tool or protocol, to attain sufficiently reliable measurements for various purposes.

## AUTHOR CONTRIBUTIONS

**Terrence D. Jorgensen:** conceptualization; methodology; writing – original draft; software; formal analysis; visualization; data curation. **Suzanne Jak:** writing – review and editing; funding acquisition.

## CONFLICT OF INTEREST STATEMENT

The authors declare no conflict of interest.

## DISCLOSURE OF THE USE OF ARTIFICIAL INTELLIGENCE‐GENERATED CONTENT (AIGC) TOOLS

No generative AI tools were used in the creation of this article.

## Data Availability

Our OSF project provides the (transformed) data and R syntax files to reproduce all reported analyses in this paper: https://osf.io/vg5uq/.

## Footnotes

^1^
This example is used to facilitate communicating many nuanced concepts in a concrete way, but it is not the only such design to which the concepts are applicable. For example, items could be replaced by raters (Lin et al., 2025; Ten Hove et al., 2022) to measure group‐level traits (e.g., independent raters observe students to measure classroom climate), or individual subjects could be the ‘cluster’ when each subject is repeatedly measured (or observed) using multiple items (or raters). For other such analogous designs, the reliability indices we define can still be applied, but different qualitative labels might be more suitable.

^2^
Our sections illustrating LMM and SEM provide more detail about the homoskedasticity assumption, and how SEM allows for heteroskedasticity.

^3^
As mentioned in Footnote 1, each of these indices is applicable in other analogous designs where the target is at Level 2 and fully crossed with a facet of generalization, but other qualitative labels might be more suitable. For example, if items are replaced by raters (a $\left(s:c\right)\times r$ design; Ten Hove et al., 2022; Lin et al., 2025) to measure cluster‐level traits, our inter‐item reliability coefficients should instead be labelled IRR. In the case of repeatedly measured subjects (who are then the ‘clusters’), our IRR should instead be labelled as an index of stability or test–retest reliability (Brennan, 2001, p. 112).

^4^
This is more commonly referred to as ‘scale reliability’, or specifically as ‘composite reliability’ (coefficient ω), limiting the appropriateness of those terms when quantifying reliability of a single item, as we do here using GT. The term ‘internal consistency reliability’ is also frequently used, particularly to describe what is quantified by coefficient α, which is also more limited than what GT allows us to quantify (i.e., not only consistency but absolute agreement). Thus, for the remainder this paper, we follow the recommendation of an anonymous reviewer to refer to this as ‘inter‐item’ reliability, analogous to IRR.

^5^
In our SEAS example, a numeric criterion might be used if a school must meet a threshold to qualify for funding—for example, for organising workshops to improve parental involvement in students’ academic success. A cut‐score‐specific D‐coef can be reported by indicating the cut‐score about which agreement is evaluated—for example, a cut score of ξ=4 could be reported as ${\text{ICC}}_{s}(\text{A}=4,n_{t:s},n_{i})$.

^6^
Dependability increases as more extreme cut scores are used for classification. We refrain from further discussion of cut‐score D‐coefs because manipulating the cut score does not help illustrate how the measurement protocol might be improved. Furthermore, the IRT literature has shown that conditional reliability (Nicewander, 2018) is typically lower for more extreme scores, casting doubt on the gain in dependability by classifying based on more extreme scores.

^7^
G‐Theory literature (Brennan, 2001; Cronbach et al., 1972; Shavelson & Webb, 1991) refers to this as a *decision study* (D‐study), often using $n^{\prime }$ to indicate the hypothetical sample size of a facet, in place of the observed sample size n from the generalizability study (G‐study) used to estimate the variance components.

^8^
Only variance that reflects between‐school differences (i.e., $\sigma_{ts}^{2}$) should be included in the numerator and denominator of a consistency index. Unfortunately, the nesting of teachers within schools does not allow disentangling pure teacher effects ($\sigma_{t}^{2}$) from the confounded variance component $\sigma_{t:s}^{2}=\sigma_{t}^{2}+\sigma_{ts}^{2}$, so any $\sigma_{t}^{2}>0$ cannot be excluded from the numerator or denominator.

^9^

https://osf.io/vg5uq/.

^10^
Although the parameters have equivalent interpretations, estimates can differ negligibly between LMM and CFA due to maximizing different likelihoods (Jorgensen, 2021; Vispoel et al., 2023): the former univariate, the latter multivariate (items stored in wide format).

^11^
Upon rejecting a $H_{0}$ of full metric invariance, the interpretability of factors could be preserved by establishing partial invariance—for example, by testing individual indicators for differential item functioning (DIF). However, interpreting the meaning of different loadings across levels remains difficult. Violations of metric invariance across clusters actually implies that Level‐1 loadings should be specified as random parameters that vary across Level‐2 units, which would imply cluster‐specific reliabilities. This methodology needs further exploration and lies beyond the scope of this paper.

^12^
CFA models for continuous indicators include three types of measurement parameters: intercepts, factor loadings, and residual variances. Parallel indicators are assumed to be homogeneous with respect to all three types of parameter, whereas essentially parallel indicators only assume equal factor loadings and residual variances. The intercepts in Figure 2 can vary across items, reflecting the essentially parallel assumption.

^13^
Similar to the parallel assumption described in Footnote 12, full versus essential tau‐equivalence can be distinguished by whether intercepts are also assumed equal across items.

^14^
Our supplementary R syntax on OSF shows that our data provide ample evidence to reject this $H_{0}$, $\chi^{2}(9)=99.071,p<.0001$.

^15^
It is well‐documented that the famous CTT coefficient α (Cronbach, 1951) is equivalent to model‐based reliability when a one‐factor model imposes tau‐equivalence constraints, although α was derived without assuming an underlying factor model (Cronbach, 1951; Sijtsma, 2009; Sijtsma & Pfadt, 2021).

^16^
Ten Hove et al. (2022, Design 1, pp. 653–654) proposed IRR coefficients for a multilevel design comparable to the one under discussion here—(s:c)×r—one where the raters r are independent observers of subjects s nested within clusters c (e.g., evaluators of student–teacher relationships who observe multiple students per teacher). Whereas we propose ICCs for shared constructs, Ten Hove et al. (2022, table 3) proposed cluster‐level ICCs for configural constructs that are analogous to those of Geldhof et al. (2014), but with raters (fully crossed with the nested facets) interchanged with items. Thus, all discussion of Geldhof et al.'s (2014) coefficients also applies to those of Ten Hove et al. (2022).

^17^
Lin et al. (2025) raised an analogous critique of cluster‐level ICCs proposed by Ten Hove et al. (2022). Just as Ten Hove et al. (2022, for raters) and Geldhof et al. (2014, for items) proposed analogous hypothetical cluster‐level reliability coefficients (for latent components; see Footnote 16), Lin et al. (2025, equations 19 and 20) proposed valid reliability formulas for observable composites of (crossed) rater responses about subjects within clusters, and their index of consistency is analogous to the reliability formulas proposed by Lai (2021) for composites of item responses about clusters by their members (nested raters).

^18^
Our G‐coef and D‐coef are analogously equivalent to the manifest cluster‐level reliability coefficients proposed by Lin et al. (2025, equations 19 and 20), when replacing items by independent raters of the subjects within clusters (where raters are crossed with clusters, rather than nested within clusters as in our motivating example).

^19^
It is worth noting that our online supplementary R syntax provides some evidence against this assumption, in that a likelihood ratio test showed significantly better fit without equating loadings across levels, $\chi^{2}(3)=10.76,p=.013$, indicating it might be informative to probe which item’s loadings vary across schools.

^20^
As a reviewer pointed out, even if two such factors were able to be extracted in an EFA, one would struggle to interpret them as being different factors at all. This is the point we made in the previous section on Substantive Interpretation.

^21^
Other identification methods would be equivalent, such as our congeneric example: by estimating both factor variances under the constraint that they sum to 1 (so the between‐level factor variance is ICC). Alternatively, the within‐level factor's variance can be fixed to $\psi_{1,1}^{\text{(W)}}=1$, allowing the between‐level factor variance $\psi_{1,1}^{\text{(B)}}$ to be freely estimated because its loadings are equivalent to within‐level loadings (i.e., Stapleton & Johnson, 2019, also imposed metric‐invariance constraints in their illustrative example).

^22^
Note that the exact degree of attenuation involves more complicated formulae for partial‐correlation or multiple‐regression coefficients, or for coefficients in multilevel models (e.g., Cho & Preacher, 2016).
